# Portuguese wild grapevine genome re-sequencing (*Vitis vinifera sylvestris*)

**DOI:** 10.1038/s41598-020-76012-6

**Published:** 2020-11-04

**Authors:** Miguel J. N. Ramos, João L. Coito, David Faísca-Silva, Jorge Cunha, M. Manuela R. Costa, Sara Amâncio, Margarida Rocheta

**Affiliations:** 1 LEAF (Linking Landscape, Environment, Agriculture and Food) Research Center, Instituto Superior de Agronomia, Universidade de Lisboa, Tapada da Ajuda, 1349-017 Lisbon, Portugal; 2 Instituto Nacional de Investigação Agrária E Veterinária, Quinta d’Almoinha, 2565-191 Dois Portos, Portugal; 3 Plant Functional Biology Centre, Biosystems and Integrative Sciences Institute, University of Minho, 4710-057 Braga, Portugal

**Keywords:** Plant sciences, Plant domestication, Plant genetics

## Abstract

The first genome of *Vitis vinifera vinifera* (PN40024), published in 2007, boosted grapevine related studies. While this reference genome is a suitable tool for the overall studies in the field, it lacks the ability to unveil changes accumulated during *V. v. vinifera* domestication. The subspecies *V. v. sylvestris* preserves wild characteristics, making it a good material to provide insights into *V. v. vinifera* domestication. The difference in the reproductive strategy between both subspecies is one of the characteristics that set them apart. While *V. v. vinifera* flowers are hermaphrodite, *V. v. sylvestris* is mostly dioecious. In this paper, we compare the re-sequencing of the genomes from a male and a female individual of the wild *sylvestris*, against the reference *vinifera* genome (PN40024). Variant analysis reveals a low number but with high impact modifications in coding regions, essentially non-synonymous single nucleotide polymorphisms and frame shifts caused by insertions and deletions. The sex-locus was manually inspected, and the results obtained are in line with the most recent works related with wild grapevine sex. In this paper we also describe for the first time RNA editing in transcripts of 14 genes in the sex-determining region, including *VviYABBY* and *VviPLATZ*.

## Introduction

*Vitis vinifera vinifera* (hereafter *vinifera*) is a worldwide important crop, mainly due to wine production. The first *Vinifera* genomes versions^1,2^, were obtained from PN40024 genotype, a Pinot Noir^1^ variety back crossed to reach 93% homozygosity, back in 2005. In the recent past, the *Vitis* genus has received more attention and the sequences of more varieties were made available^3–10^. Although the contribution of these *vinifera* genomes is out of question for the present knowledge, they are limited in providing information on particular changes that occurred during domestication. One of the most relevant subspecies of *V. vinifera* history is *Vitis vinifera sylvestris* (hereafter *sylvestris*), which some authors have referred as *vinifera*^11,12^. *Sylvestris*, free from human selection, may provide clues to explain *vinifera* domestication. This human intervention was an economically important step for grapevine, responsible for morphological changes that include larger berry and bunch size, higher sugar content, altered seed morphology, and a shift from dioecy to a hermaphroditic mating system^13^. The dioecious *sylvestris*, produce female individuals with reflexed stamens, where infertile pollen is produced, whereas male individuals are unable to produce grapes as flowers lacks a functional carpel^14^.

In the *Vitis* genus, several studies have contributed to partially uncover the genomic regions that may be responsible for the differences in flower development between hermaphrodites and dioecious plants^15–17^. However, the identified regions (chr2:4,907,434.0.5,050,616)^15,16^ are poorly sequenced, assembled and annotated in the canonical reference genome^1,2,11,13,17^. Recently, two new studies^18,19^ suggested the possible players involved in *sylvestris* sex specification by uncovering *INAPERTURATE POLLEN1* (*INP1*) as a male sterility gene and proposing a set of genes responsible for female sterility.

The understanding of all players involved in *Vitis vinifera sylvestris* dioecy is still incomplete. Events as RNA editing in nuclear transcripts have already been proven in various organisms, as humans^20–22^, cephalopods^23^, *Arabidopsis thaliana*^24,25^ and *Vitis vinifera sylvestris*^26^.

RNA editing, defined as post-transcriptional alterations of RNA molecules by insertion, deletion or modification, not including processes as splicing, capping or polyadenylation^20^, may also contribute to sex differentiation in *V. v. sylvestris*.

The aim of this paper is to compare the genomes of *sylvestris* against PN40024 and establish the differences that can highlight the main domestication events. To achieved the proposed aim, we defined four major tasks: (1) to characterize the re-sequencing of *sylvestris*, considering two distinct individuals, one with male flowers and a second one with female flowers; (2) to compare in detail the genomes of the *sylvestris* individuals to the reference genome, in terms of Single Nucleotide Polymorphisms (SNPs) and Insertions/Deletions (InDels); (3) to compare the different alleles found for each *sylvestris* specimen, focusing on chromosome 2, where the sex-determining region has been proposed to be present; (4) to access RNA editing events in genes associated to the sex-determining region.

## Results

**Figure 1:**
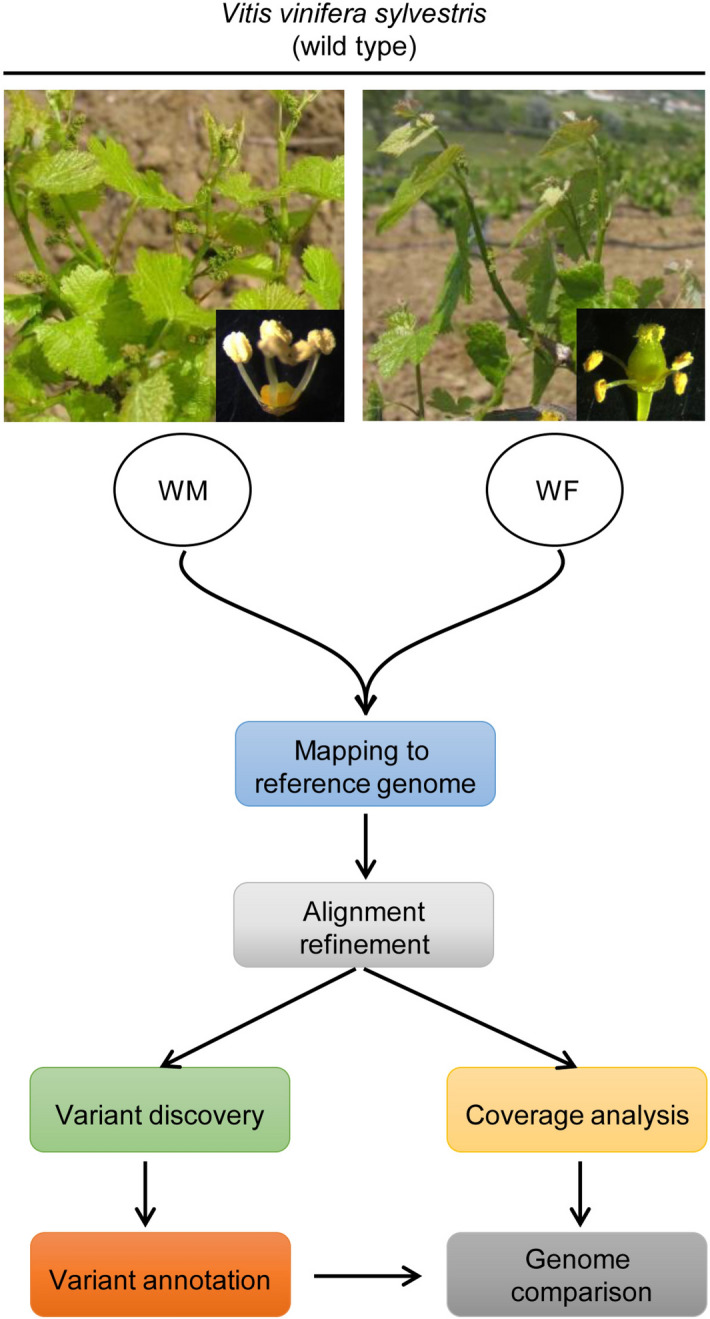
Sampling material of *Vitis vinifera sylvestris* (wild type) and flowchart of data analysis steps. WM individual show carpel abortion (typical of a male flower type); WF individual has reflexed stamens (representing a female flower type). Reference genome is PN40024 (CRIBI, 12X).

The genomes of two wild-type individuals (*sylvestris*) were obtained by Illumina (2 × 125) and the reads were mapped against the reference genome of *vinifera* PN40024^1,2^ (Fig. 1). The reference genome used in this study was firstly published in 2007 after controlled breeding of Pinot Noir, creating a lineage with about 93% homozygosity^1^. This genome (8X coverage) consists in 487 Mb spread into 3,514 scaffolds (N_50_ = 2.07 Mb)^1^. Scaffolds were further associated within the 19 chromosomes of *Vitis* plus 13 virtual chromosomes, corresponding to 12 random chromosomes (associated to the real chromosomes 1, 3, 4, 5, 7, 9, 11, 12, 13, 16, 17 and 18) and a completely unknown chromosome, where sequences that were not fully associated with a real chromosome were deposited^1^. The sequenced genome was further improved with a 12X coverage and subsequent gene annotations were published^1,2^.

### The majority of the wild-type reads map to the reference genome

Around 97% of the high-quality reads obtained were successfully mapped to the reference genome: 478,309,356 reads in total (Table 1). After refinement steps (please see Materials and Methods for details), 92% of the mapped reads were kept. From those, 89% mapped only once to the reference genome (Table 1). About 10% of the mapped reads lost their pair in the process or mapped onto a different chromosome (Table 1).

**Table 1:** Number of wild-type reads mapped against the reference genome (PN40024).

Read category	WF	WM	Total
Total mapped reads	209,622,690	268,686,666	478,309,356
Unique	187,424,438 (89.41%)	240,250,914 (89.42%)	427,675,352 (89.41%
Non-unique	22,198,252 (10.59%)	28,435,752 (10.58%)	50,634,004 (10.59%)
Singletons	1,782,319 (0.85%)	2,151,281 (0.80%)	3,933,600 (0.82%)
Cross-contigs	20,897,087 (9.97%)	24,790,708 (9.23%)	45,687,795 (9.55%)

Unique: reads that map only to one site on the reference genome. Non-unique: reads that map to multiple sites of the reference genome. Singletons: reads that map to the reference genome, without mate pair. Cross-contigs: reads whose pair map to a different reference chromosome. Percentages: percentage of each class to the total mapped reads.WF: Reads obtained from the female wild individual. WM: Reads obtained from the wild type individual with male flower types.

### Comparison between the wild-type and reference genomes

**Figure 2:**
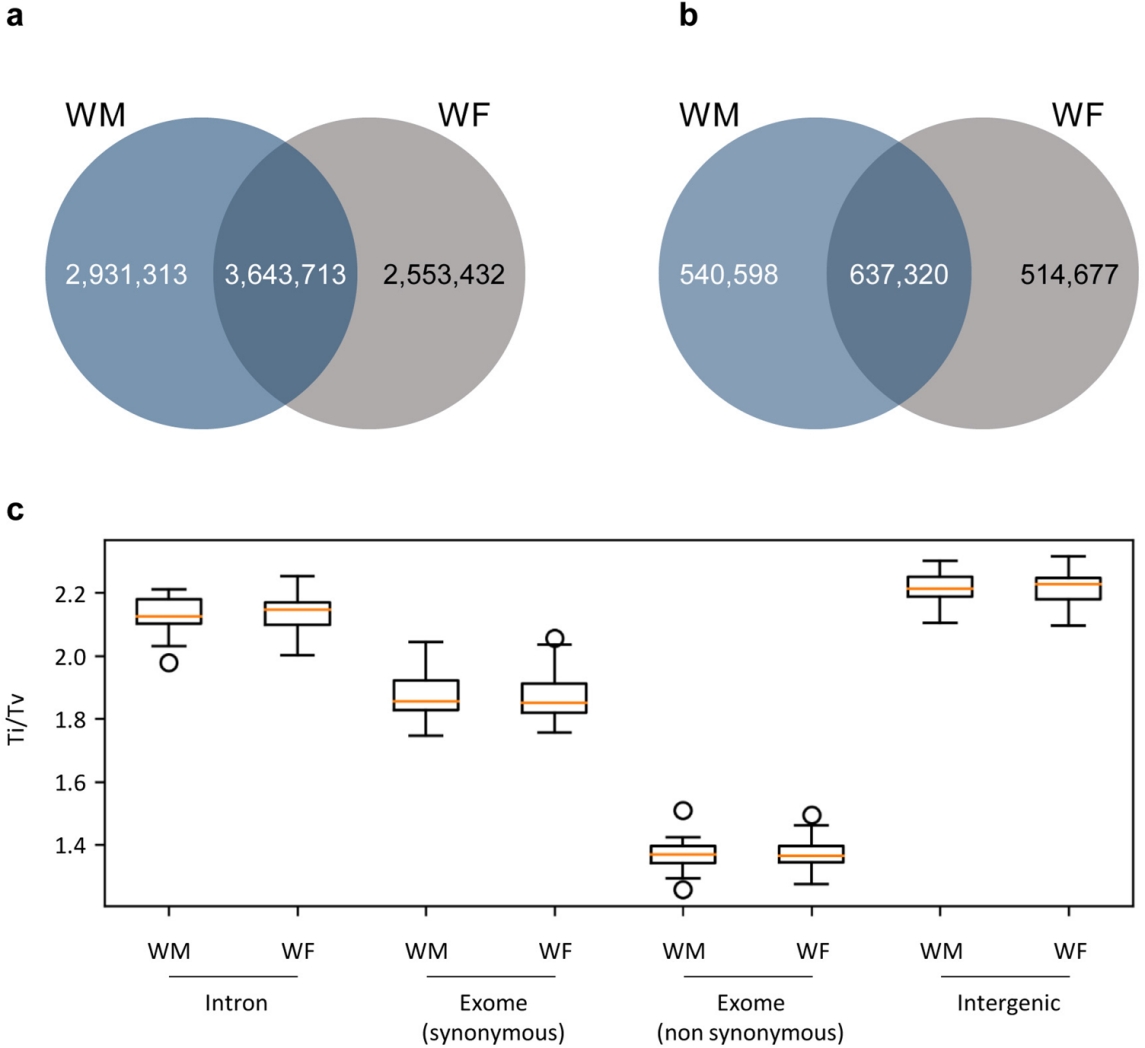
Landscape of SNPs and InDels in the two wild-type genomes. (**a**) Venn diagrams showing the number of SNPs. (**b**) Venn diagrams showing the number of InDels. Comparison was performed against the reference genome (PN40024, CRIBI, 12X). (**c**) Transition over transversion ratio (Ti/Tv) of WM (male) and WF (female) individuals according to the genomic region. For exomes, analysis was split into synonymous and non-synonymous SNPs.

Single Nucleotide Polymorphism (SNP) and Insertions/Deletions (InDels) analysis were performed between the sequences obtained for the two wild-type individuals and the reference genome. Wild-type individuals used on this re-sequencing project present distinct flower characteristics. One of the plants show male characteristics (WM), with no functional carpel and the other exhibits female flowers (WF) with a complete carpel, but with reflexed stamens producing infertile pollen. Both of these individuals had almost the same number of SNPs identified (6,197,145 for WF and 6,575,026 for WM individual), and shared half of the occurrences (3,643,713 SNPs; Fig. 2a). The InDels analysis showed the same tendency as the previous results: the number of occurrences in both individuals were similar (1,151,997 for WF and 1,177,918 events for WM) and about half of them were shared (637,320 events; Fig. 2b).

The ratio between the numbers of transitions over transversions (Ti/Tv) is usually calculated in order to assess the quality of an assembly or SNPs calling. As Ti/Tv ration has been reported dependent of the genomic context of the SNPs^27^, we have evaluated Ti/Tv on introns (ratio 2.1), exons with synonymous SNP (1.9), exons with non-synonymous SNPs (1.4) and intergenic regions (2.2) (Fig. 2c). Our analysis confirmed that Ti/Tv was dependent of the genomic location, but not on the individuals under study, since WM and WF presented the same Ti/Tv pattern (Fig. 2c).

**Figure 3:**
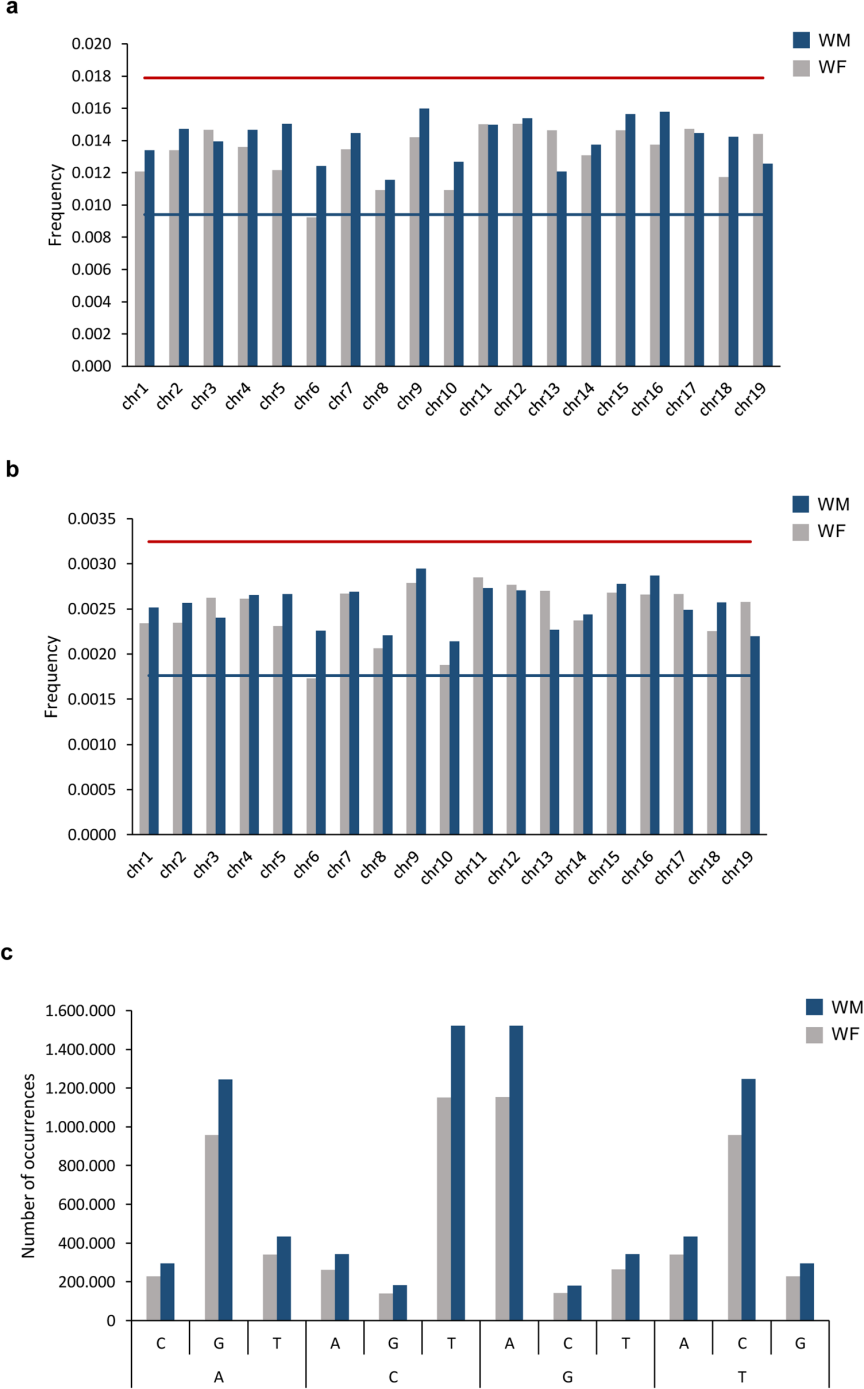
Analysis SNPs distribution and dynamic. (**a**) Frequency of events per chromosome length (base pairs) in considering SNPs and (**b**) InDels. Red and blue lines represent the Tukey's outlier fences (k = 1.5). (**c**) Number of transversions (A ↔ C, A ↔ T, C ↔ G and G ↔ T) and transitions (A ↔ G and C ↔ T). Reference: The nucleotide in the reference genome (PN40024, CRIBI, 12X); *V. v. sylvestris*: the nucleotides identified in WM and WF genomes.

The distribution of SNPs and InDels, on the genome, was evaluated for each chromosome, by calculating the frequency of occurrences (number of SNPs/InDels by chromosome size). In both SNPs and InDels, the chromosome 6 was the one with the lowest number of events. For this chromosome, 1.00% of the bases were found to be changes on a single nucleotide (frequency of 0.011; Fig. 3a and Supplementary Figure S1) and InDels were identified in 0.20% of the chromosome (frequency of 0.0020; Fig. 3b and Supplementary Figure S1). The low number of modifications in chromosome 6 is essentially caused by WF individual, which presents a fraction of SNPs below Tukey ‘s fence. The least conserved chromosome was the 9 with a percentage of SNPs of 1.5% (frequency 0.015; Fig. 3a and Supplementary Figure S1) and InDels covering 0.29% of this chromosome size (0.0029; Fig. 3b and Supplementary Figure S1). The modification enrichment in chromosome 9 is mainly due to the WM individual. The pairwise comparison between WM and WF, besides chromosome 6 distinct profiles between the two tested individuals, as referred, also highlights the higher number of modifications in WM chromosome 13 than in WF (Supplementary Figure S2).

The analysis of chromosome regions with higher and fewer number of modifications, carried out in both individuals, showed that the density varied along the chromosomes, however most of them do not have a specific location with high density of SNPs and InDels simultaneously (Supplementary Figure S3).

We analysed the nature of the substitutions in SNPs by comparing the nucleotides on the reference genome with the nucleotides observed for the wild type. In accordance with other studies^10,12^, the number of transitions (substitution between adenosine and guanine or cytosine and thymine) were much more frequent on both genomes than transversions (substitutions between adenosine and cytosine, adenosine and thymine, cytosine and guanine or guanine and thymine) (Fig. 3c and Supplementary Figure S4).

**Figure 4:**
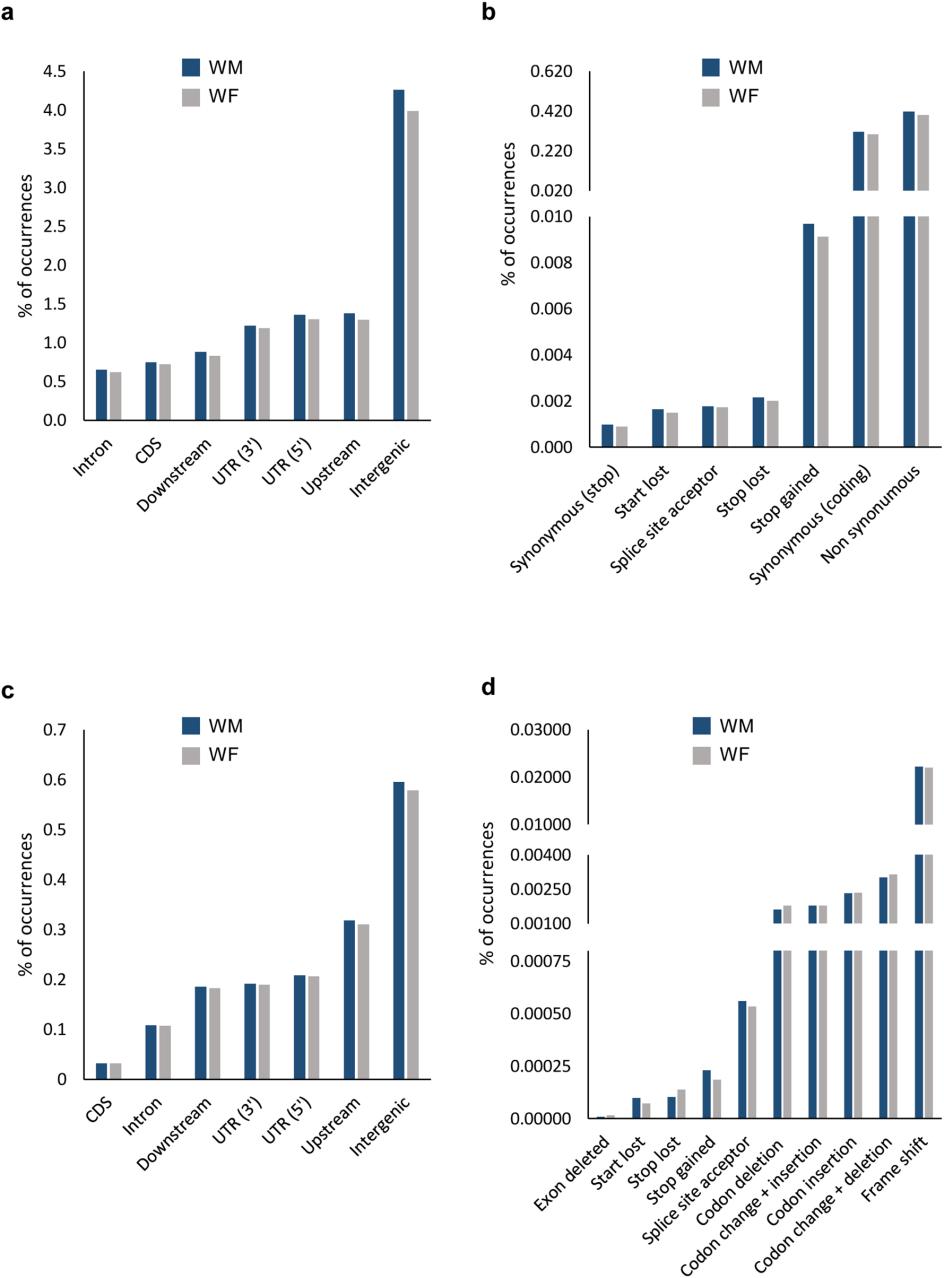
The gene context of the identified modification in wild-type genomes (WM and WF). (**a**) Percentage of regions affected by SNPs and (**b**) the impact caused by the SNP when it occurs in CDS regions. (**c**) Percentage of features affected by InDels and (**d**) its impact in CDS regions. Reference genome and annotation: PN40024, CRIBI, 12X V2.1. For events between genes, the most relevant context was chosen (e.g. upstream instead of downstream), as described by SnpEff documentation. Upstream and downstream were considered for a 5 kbp range.

The effects of SNPs on protein-coding regions were also investigated by analysing the SNP location within gene context and the impact caused. To perform this analysis, we have calculated the percentage of modified features. As expected, the most affected context by SNPs was the intergenic (about 4.1% of intergenic sites reveal differences compared to reference genome; Fig. 4a and Supplementary Figure S5). Upstream, UTR regions and downstream are also affected regions (ranging from 1.3 to 0.9%). Introns and CDS regions reveal almost the same levels of SNPs, in terms of percentages (0.6% and 0.7%, respectively). When SNPs affected CDS, we further studied the in silico impact expected (Fig. 4b and Supplementary Figure S5). The percentage of CDS modifications with synonymous and non-synonymous impact was almost the same (0.3% and 0.4%, respectively). A high percentage of premature STOP codons was found, essentially considering that this impact is among the most disruptive ones (0.01%).

The overall distribution of InDels is almost identical to SNPs: the majority of indels occurs in intergenic (0.6%), followed by upstream, UTRs and downstream regions (ranging between 0.3% and 0.2%; Fig. 4c and Supplementary Figure S5). However, contrary to SNPs, only a small portion of CDS are affected by InDels (0.03%). In terms of impact (considering CDS context), the majority of InDels caused frame shift (0.02%; Fig. 4d and Supplementary Figure S5). InDels resulting in codon change + deletions, corresponding to events where at least one codon is changed and at least one is deleted (0.003%), codon insertion (0.002%), codon change with insertions and simple codon deletion (both lower than 0.002%) were also found (Fig. 4d and Supplementary Figure S5).

### Heterozygosity levels

**Figure 5:**
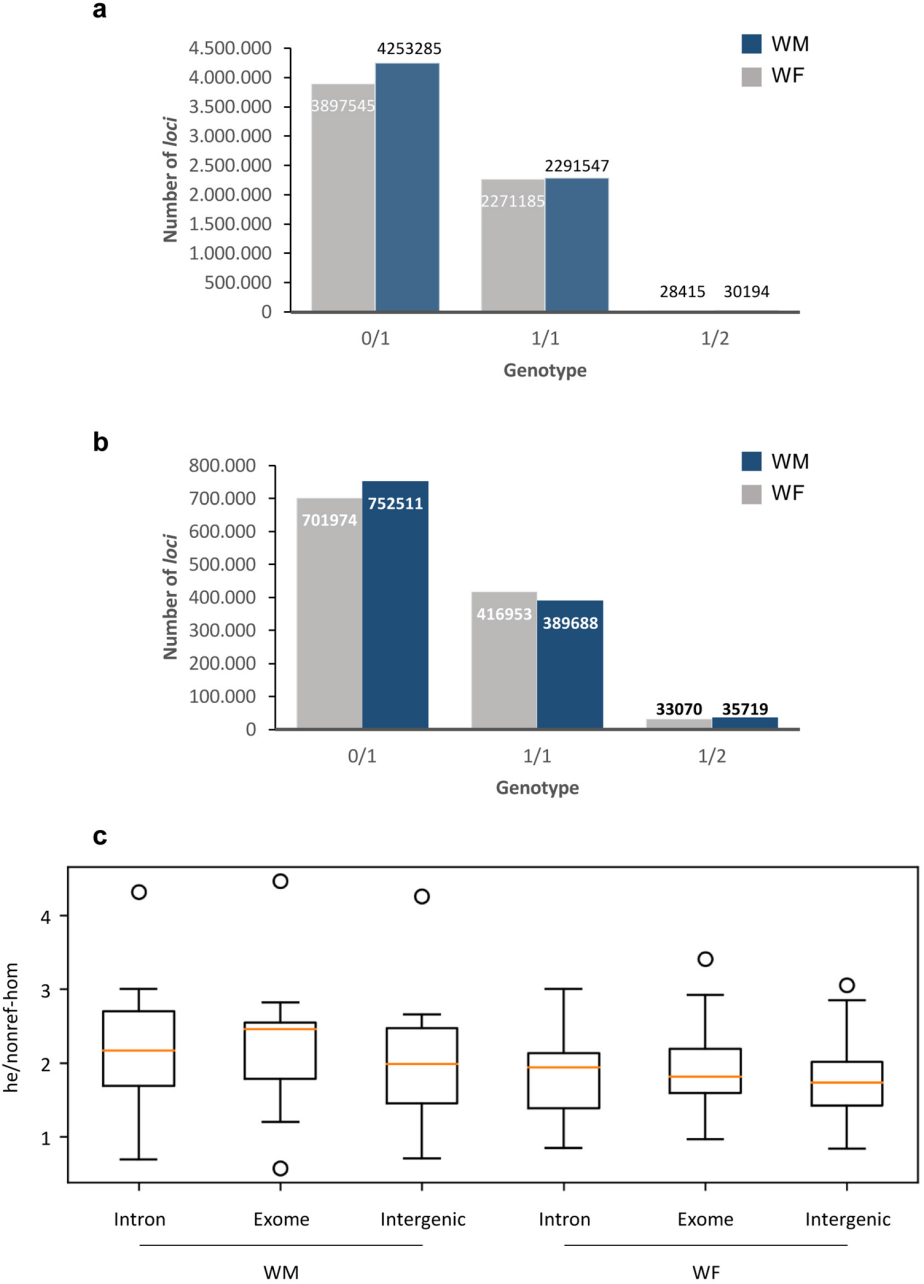
General heterozygosity analysis. Number of homozygous (1/1) and heterozygous (0/1 and 1/2) *loci* evaluated for the wild type re-sequenced genomes. (**a**) Evaluation according to SNPs. (**b**) Evaluation according to InDels. (**c**) Heterozygous over non-reference homozygous ratio (he/nonref-hom) for each individual (WM and WF) according to multiple genomic locations. 0/1 represents *loci* where two alleles were observed, one being identical to reference genome (PN40024, CRIBI, 12X); 1/1 represent *loci* where only one allele was observed, being different than reference; 1/2 represents heterozygous *loci*, being both alleles different than reference.

The nature of the SNPs and InDels was evaluated regarding their homozygosity. Most of the events were heterozygous (0/1 and 1/2) (Fig. 5 and Supplementary Figure S6). The *sylvestris* individuals had about 4,104,719 heterozygous SNPs and 761,638 heterozygous InDels, being the WM plant more heterozygous than the WF (Fig. 5). The number of homozygous alterations between *sylvestris* and the reference genome (1/1) was much lower (2,281,366 homozygous SNPs and 403,321 homozygous InDels) (Fig. 5 and Supplementary Figure S6). Combining SNPs and InDels, the total number of heterozygous *loci* (0/1 and 1/2) was 4,866,357 while the homozygous (1/1) changes were 631,487. The ratio between heterozygous/homozygous SNPs was 1.80 and1.89 for InDels.

An analysis of the heterozygous (0/1 and 1/2) over non-reference homozygous (1/1) SNPs, considering the *sylvestris* individuals separated, at multiple genomic locations, revealed slightly differences between them (Fig. 5c). Ratios obtained were 2.2 for WM (introns and exons), 2.0 for WM (intergenics), 2.0 for WF (exome) and 1.8 for WF (introns and intergenics) (Fig. 5c). When considering exons, the ratios obtained for synonymous and non-synonymous were the same (data not shown).

### Validation of genome sequencing by Sanger analysis

Some of the SNPs detected on the presented comparison were validated by Sanger sequencing, targeting modifications in four different genes. From the nine modifications tested, four were homozygous and shared by both individuals, suggesting that these modifications are exclusive of wild-type individuals (Supplementary Figure S7a, b and c). One of the modifications shared by both individuals represented a case where the wild type genomes are heterozygous (Supplementary Figure S7d). We also validated the occurrences of individual exclusive modifications, where one of the individuals (WF) was homozygous and the other (WM) was found to be heterozygous (Supplementary Figure S7d). All the modifications identified in Sanger analysis were in accordance with Illumina results. Although, a higher number of changes were detected than those presented in the Supplementary Figure S7, the ones presented are an example to validate the results.

### De novo assembly metrics

A de novo assembly of the genomes was achieved to better assess the sex-determined region. All the reads obtained in this project were cleaned to remove low confidence reads. The female and male assembly used 224,542,530 and 287,279,296 reads, respectively (Table 2). The coverage estimates for this assembly was 58X to female and 74X to male individual (Table 2). After the assembly, the female genome was comprised into 102,650 contigs with a total size of 364,897,899 bp (N50 of 5,795), whereas the male was represented by 108,550 contigs, totalling 370,798,370 bp (N50 of 5,317; Table 2). A %CG ratio of 34 was identified for both samples under study.

**Table 2:** Parameters related to de novo assembly of the wild female (WF) and male (WM) individuals. Total number of reads: the number of reads obtained from the sequencing. Reads used on the assembly: number of reads after cleaning with BBDuk (BBMap package). Coverage: Expected coverage, considering the reference genome of 486 Mbp. Number of contigs: The amount of contigs obtained from the assemblies with more than 1kbp. Total assembly size: The total size (in base pairs) of the obtained genome. N50: The size of the shortest contig at 50% of the total genome length. %CG: Percentage of Cytosine and Guanines over the total genome length.

De novo assembly parameters	WF	WM
Total number of reads	236,749,964	302,723,774
Reads used on assembly	224,542,530	287,279,296
Coverage	58X	74X
Number of contigs	102,650	108,550
Total assembly size (bp)	364,897,899	370,798,370
N50	5,795	5,317
%CG	34	34

### Manually inspection of chromosome 2

Recently, knowledge about the sex-determining region was updated^18,19^, and the 24 genes located in the sex-determining region were manually inspected from a de novo assembly performed with the raw data obtained for both male (WM) and female (WF) *sylvestris* individuals. The number of contigs identified for each one of these genes was listed in Supplementary Table S1. Both studies^18,19^ indicate that the gene coding for *INAPERTURATE POLLEN1* (*VviINP1*) is responsible for male sterility, due to an 8-bp deletion in the CDS region, detected in female individuals, causing a premature stop codon. We found that this gene has homology with VIT_200s0233g00051 and VIT_202s0154g00120, associated with chromosomes “unknown” and 2, respectively. We consider that these two IDs are two alleles of the same gene. In the samples tested under this re-sequencing project, we identified the two alleles in the male individual, but the female individual only has the allele with the 8-bp deletion (VIT_202s0154g00120). Although there was a consensus on the identification of *VviINP1* as responsible for male sterility, the results for a female sterility gene were not yet unanimous and several genes were proposed as responsible for this trait. The most promising gene reported for female sterility were *VviYABBY* (VIT_202s0154g00070) and *VviPLATZ* (VIT_202s0154g00150)^18^. As far as we were able to identify, both of these genes only presented one allele in the hermaphrodite reference genome. In our wild plants the genes were heterozygous in the male and homozygous in the female plants.

**Figure 6:**
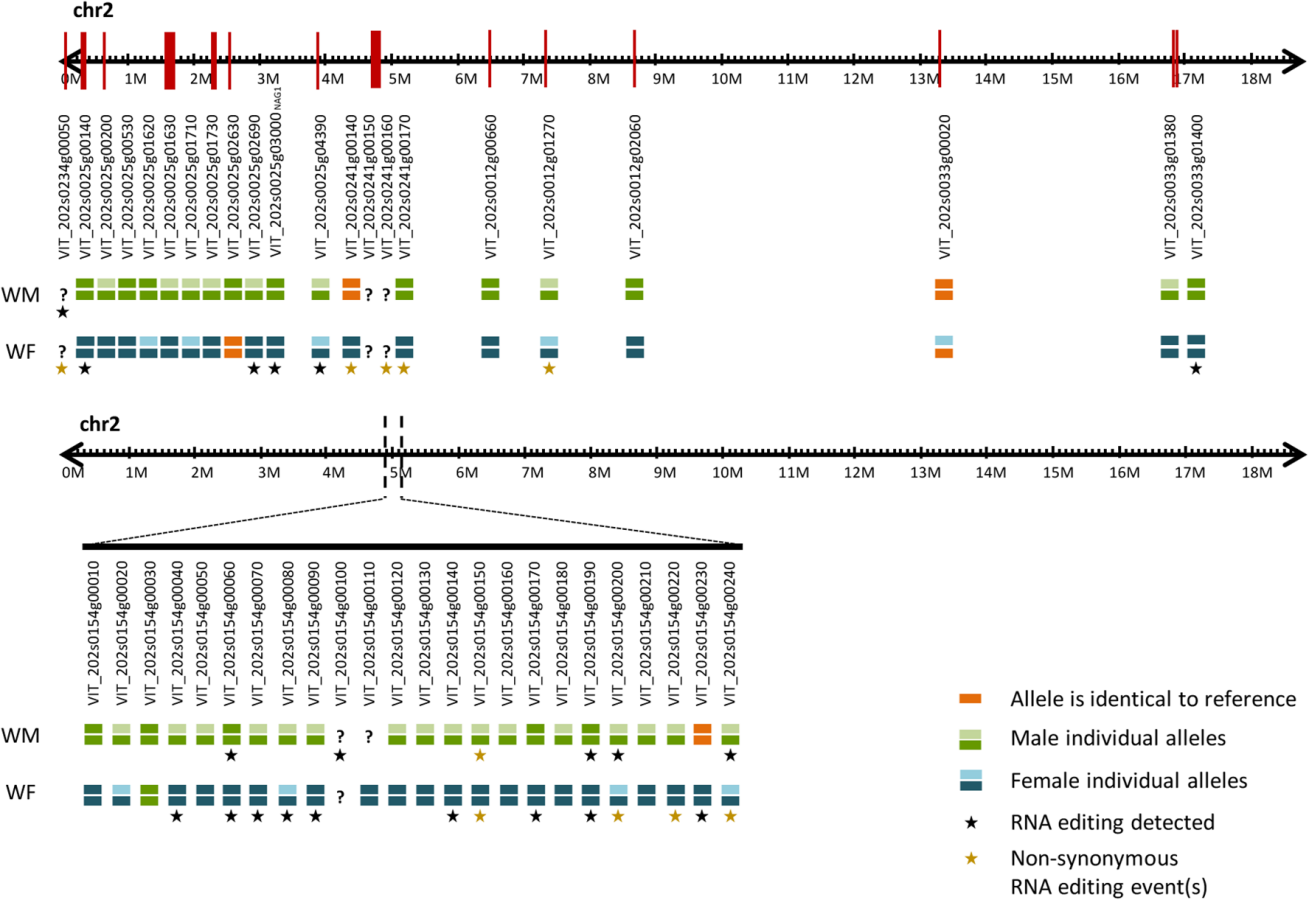
Representation of location and differences between the most relevant genes and sex-associated *loci* in chromosome 2 after manual validation. Top panel represents genes in chromosome 2, with putative impact on sexual structures according to literature; bottom panel represents genes in the sex-determining region (chromosome 2). Orange bars represent alleles of the de novo assembled genomes identical to the reference (PN40024, CRIBI, 12X). Green bars represent alleles identified in the male individual. Blue bars indicate distinct alleles observed in the female individual. When a male and a female allele were identical, the same colour was used (green). Question marks refer to genes with low assembly resolution whose sequence was not identified correctly. Stars indicate genes where RNA editing was identified in transcripts, detected by comparing the genome sequences against the previous published transcriptomes, for the same individuals.

We also manually inspected several genes on chromosome 2, some of them described to be involved in flower development like *VviNGA1* (VIT_202s0025g03000)^30^ and *VviSLK2* (VIT_202s0025g04390)^31^. Other 20 genes located on chromosome 2, with distinct transcription patterns were previously^15^ detected^16^ and were subject to this analysis, in a total of 46 genes. From the 46 genes analysed, the male individual studied was heterozygous for 24 genes (Fig. 6). The alleles of three of WM genes were identical to the reference genome (VIT_202s0241g00140, VIT_202s0154g00230, VIT_202s0033g00020; Fig. 6). In the female individual, 9 out of 46 genes were heterozygous with both alleles presenting differences compared to the reference genome; only two of 46 genes had alleles identical to the reference genome (VIT_202s0025g02630 and VIT_202s0033g00020); and the remaining 36 genes were homozygous with differences when compared to the reference. Also, we found only one identical gene between male and female individuals (VIT_202s0154g00030) being homozygous, but distinct to the reference genome (Fig. 6).

### RNA editing was found in transcripts of the sex-determining region

RNA editing differences, defined as differences found in the RNA after transcription, including nucleotides substitutions, were identified in *Vitis*^26^, using these individuals and previous transcriptomic data^32,33^. It is relevant to report here, that no RNA editing was found for *VviINP1* accessions (VIT_200s0233g00051 and VIT_202s0154g00120). We found RNA editing in the 3′ UTR transcripts of *VviYABBY* (chr2:4,864,628), in female flower at developmental stage D, where a thymine was replaced by an adenosine in some transcripts (mRNA two-variants) (Supplementary Figure S8a). In WM (chr2:4,864,509) we identified and validate by Sanger sequencing a mRNA two-variants modification in developmental stages D and H (Supplementary Figure S9a). We also found RNA editing in *VviPLATZ* transcripts, where female and male inflorescences show different RNA editing patterns: the female only presents one event (chr2:4,950,043) (Supplementary Figure S8a), and the male plant has six events (chr2:4,950,184; chr2:4,950,199; chr2:4,950,364; chr2:4,950,393; chr2:4,950,435 and chr2:4,950,443), totalling seven RNA edited *loci* (Supplementary Figure S8b). From these seven *loci*, only two (both in male plants) are not in the coding region (chr2:4,950,184 and chr2:4,950,199) (Supplementary Figure S8b). The in silico analysis revealed seven genes in which RNA editing (six in SDR and one outside) was found in WM but only *VviPLATZ* showed a non-synonymous modification. In WF, nine out of twenty-three genes with RNA editing showed non-synonymous modification, which includes *VviPLATZ* and *VviFSEX* (VIT_202s0154g00200) (Fig. 6).

To confirm that RNA editing is not an exclusive event in these two individuals (WM and WF), we performed a second validation using a distinct female plant (WF_2). This validation targeted VIT_202s0033g01400, a gene in chromosome 2 far from the sex-determining region. We found several editing events, some with the two forms of mRNA, edited and unedited and one full edited event (Supplementary Figure S9b). The RNA editing tested by the Sanger sequencing performed on the WF_2 individual, revealed the same changes detect by NGS for WF, suggesting that RNA editing is not an exclusive event of WF and WM individuals.

Modified mRNAs were found inside and outside the sex-determining region, with distinct profiles between male and female individuals (Fig. 6).

The results reported in this paper, provided a comparison between the reference genome (*vinifera*) and *sylvestris*. We have identified SNPs and InDels between the two subspecies and investigated the sex *locus* region, providing a further evidence of which genes may be key players in sex specification of grapevine. We have also found RNA editing in genes belonging to the sex-determined region, namely *VviYABBY* and *VviPLATZ*.

## Discussion

The analysis of the wild-type individuals (*V. v. sylvestris*) reveals a high similarity to PN40024, used as reference genome (*V. v. vinifera*). In fact, 89% of the sequences maps only once to PN40024 genome. 10% of the reads whose pair was lost or map to a different chromosome may be due to the remaining heterozygosity present in the reference genome (93% homozygous^1^), namely, due to the 13 virtual chromosomes created, where heterozygous sequences may reside^1,2^, or other repetitive regions as transposable elements or telomeres, among others. As referred above, we have identified an average of 4,104,719 heterozygous SNPs and 761,638 heterozygous InDels. A similar study from 2017^10^ comparing four grapevine cultivars obtained heterozygous SNPs ranging between 178,650 and 934,946 and heterozygous InDels of 16,768–47,852. Discrepant values between cultivars and *sylvestris* plants, had already been reported considering only deleterious alleles^11^. The higher number obtained in our study suggests a large distance between wild-type individuals (*sylvestris*) and the domesticated grapevine (*vinifera*). The ratios heterozygous/homozygous are in the same range as calculated by Mercenaro et al. (2017)^10^, and between the findings reported by Zhou et al. (2017)^11^ (~ 77%) and D’Onofrio (2020)^34^ (~ 40%).

It is described that domestication pressure set on cultivated varieties, improving grape characteristics, affected all chromosomes in a similar way^11,35^. In the present study, we were unable to detect a particular chromosome with significantly different number of modifications, measured by a single nucleotide change or Insertion/Deletion. Nevertheless, chromosomes 6 and 9 were highlighted as the ones with fewer and more alterations, respectively, but the number of modifications is still inside the Tukey's Fences used on the outlier’s analysis.

The analysis considering the two separate individuals also supports chromosome 6 as the most relevant to distinguish the WF and WM individuals, and the result for the pooled sample may have a great contribution from the WF, which seems to be particularly conserved on chromosome 6.

The chromosome 13 also contributes to distinguish WM and WF plants in terms of SNPs and InDels. We need to emphasize caution in extrapolating this result to sex, as differences observed here are putatively individual-specific. Chromosome 2, consensually associated to sex, is not highlighted by any one of these analyses.

Analysis of the two plant chromosomes revealed that SNPs and Indels are spread across all of them. There are regions with a very low number of modifications, which coincide with regions poorly sequenced in the reference genome, therefore, the correct read mapping was not possible.

Most of the modifications identified are found in non-coding regions, where the impact is not directly assessed. However, Shabalina et al.^36^ associated non-coding and synonymous SNPs to the gene expression, as these modifications may promote modifications at mRNA structure with consequences at stability and decay, or even by interfering in RNA editing. Similar trends of coding/non-coding and synonymous/non-synonymous were previously associated to wild populations, and according to Williamson et al.^37^ could be considered an evolutionary trait of the population instead of a mark associated to domestication efforts, essentially in non-domesticated species. However, coding/non-coding and synonymous/non-synonymous ratios were also proved to be associated to domestication, for example in bean^38,39^ and tomato^40^. The modifications identified in this paper, were found through a comparison between wild type specimens and a domesticated variety, and so, the domestication impact may not be completely discarded. Furthermore, a recent publication used 261 SNP markers to evaluate the genetic differences between Portuguese varieties and Portuguese wild populations (including the populations referred herein)^41^, to establish kinship relations and gene flow between wild individuals and cultivar varieties, as already reported by other studies^34,41,42^. This kind of genetic flow between wild and domesticated varieties may contribute to dilute the domestication impact in grapevine due to a systematic recap of wild genes/alleles. Considering these findings any direct conclusion or association between the SNPs and their cause, should be drawn with caution. It should be noted that Cunha et al.^41^ found no evidence of backcrossing between the domesticated and the wild genomes specifically used in this genome resequencing. Neither the evolutionary pressure on these two independent populations nor the effects of the reference genome domestication, were diluted by back-crosses, which means that both hypotheses could be simultaneously contributing to the observed differences.

One of the most unexpected result is the low Ti/Tv ratio (transitions over transversions) in exons, regardless the SNP impact on protein sequences (synonymous or non-synonymous). Ti/Tv ratios have been associated with the genomic context of the modification and in fact, we clearly observe an association between the genomic context and the ratio. However, the ratio we have obtained on exons (1.4–1.9) highly contrasts with the values reported by Wang et al.^27^ (circa 2.8) but is in line with the results identified by Vondras et al.^7^. A lower ratio as we observed, is getting closer to the ratio that should be observed if SNPs were random (0.5 as there are two transitions and four transversions). Low values of Ti/Tv were also observed in *Oryza sativa*^43^ and *Brassica rapa*^44^. It is evident and intriguing that, in plants, the higher the impact of a SNP, the lower is the Ti/Tv obtained.

The highest percentage of regions affected by modifications, are intergenic followed by upstream regions (5 kbp before a gene start). It is interesting to observe that the percentage of occurrences on the upstream regions are almost twice as the ones observed for downstream (5 kbp after gene ends), essentially as upstream regions are usually associated with the gene promotor region. When analyzing the modifications in CDS regions, we find that the majority of the events have impact on protein (corresponding to non-synonymous modifications or frame shifts). The high impact observed, suggests an intentional evolutionary direction beyond simple mutation randomness. The percentage of modifications within the CDS region is generally low, but the few ones detected are modifications with an expected biological impact. These results support the suggestion by Ramos et al.^32,33^, indicating that both subspecies (*sylvestris* and *vinifera*) are mostly similar, however, the few differences found lead to significant protein modification.

The fact that the two individuals used in this study share only half of the identified SNPs and InDels, suggests that these individuals are not parentally related. This idea is also supported by the heterozygous over non-reference homozygous SNPs whose ratio has been associated with lineages^27^. We obtained distinct het/nonref-hom ratios for each individual for the same genomic regions. Unfortunately, other *Vitis* studies^10,19^ lacks this kind of detail.

When considering SNPs and InDels in common (between the two wild individuals), we need to be caution when extrapolating the results to the entire wild population, as we are observing the pattern of only two individuals (one male and one female individuals). Nevertheless, as they were phenotypically distinct individuals (one producing male flowers and the other female flowers), the common modifications may correspond to wild-type specific characteristics.

As both individuals produce distinct flower types, some of the genetic differences found between them may be associated to sex. Curiously, chromosome 2, where the *locus* associated to sex has been reported by different studies^7–9^, has a similar number of SNPs and InDels, when compared to other chromosomes. Nevertheless, we performed a more detailed analysis of the sex-associated *locus* in chromosome 2. From the 46 genes studied on this chromosome, only one (VIT_202s0154g00030) has identical alleles in both wild-type individuals, and other gene was recently associated with male sterility (*INP1*)^18,19^, which makes relevant the question of whether or not the other 44 genes could be associated with sex. At present, we are not able to answer this question and further studies should be carried to determine the impact of these 44 genes, using more individuals of each flower type, in order to better associate each one of these genes with its putative role in flower sex determination. Additionally, the two individuals used on this resequencing project had already been tested for *VviAPRT3* and *VviFSEX*^9^ and the pattern was the expected for male and female.

Regarding the discussion of SNPs relevance, some of them located in coding regions, we need to be careful, since RNA editing has already been described as present in the nuclear transcripts of plants like *Arabidopsis*^24,25^ and *Vitis*^26^. In *Vitis*, the two genomes included in this work, were compared with previous transcriptomic data^17,18^. We need to highlight that the studied individuals were the same.

Eventual differences between DNA and RNA (RDDs) need to be considered and tested before concluding that SNPs have impact in plants. Combining the genomes published in this work and the transcriptomes already made public^32,33^, we identified 14 genes in the sex-determining region whose transcripts show RNA editing, the majority of them exclusive of the female plant (8 out of 14). *VviYABBY* (VIT_202s0154g00070) is one of those genes and presents a two-variants RNA editing in the 3′UTR region, where edited (T-to-A) and non-edited mRNAs are simultaneously detected, at one of the tested flower developmental stages (D stage). Since the two forms are present, female flowers simultaneously exhibit messenger molecules with uracil (as a thymine is encoded by the genomes of female and male individuals) and other molecules with adenosine. A second mRNA two-variants RNA editing event was detected for 3′UTR of *VviYABBY* on male individual (WM) on developmental stages D and H. Which is also interesting, is the RNA editing pattern of *VviPLATZ* (VIT_202s0154g00150), as male and female inflorescence show different RNA editing events, without overlap, including events within the CDS regions.

Considering the relevance of the CRIBI database in *Vitis* research, we need to stress the urgency of a complete curation of the genome deposited in that database. An effort should be carried out to resolve the sequences deposited in the “unknown” and “X_random” chromosomes, as these sequences usually correspond to alleles of the genes present in the remaining 19 chromosomes.

Summing up, the work described in this article contributes to the identification of putative specific modifications of *sylvestris* when compared to *vinifera*. This paper clarifies questions raised on previous studies using the transcriptome^32,33^, where different levels of gene expression were observed between *sylvestris* and *vinifera* transcriptomes. The identification of SNPs and InDels in the promotor region of the genes (5 kbp upstream of the gene) may explain the differential expression levels detected. In addition, the nucleotide differences revealed here, within coding regions, suggest that different alleles or gene duplications may have distinct importance in *sylvestris* and in *vinifera*.

## Materials and methods

Sampling and DNA extraction. Representatives from *V. v. sylvestris* (wild type) were selected from INIAV, Dois Portos (Lisbon district, Portugal, 39.041395, − 9.181956), where a wild type collection was established. One of the selected individuals exhibited female flowers (WF) and the other displayed male flowers (WM). Fresh leaves were collected from each selected individual. Leaves were grinded in liquid nitrogen and the DNA was extracted and purified with the DNeasy Plant Mini Kit (Qiagen), according to the manufacturer´s instructions. DNA quantity and quality was evaluated using a microplate reader Synergy HT (Biotek, Germany), using the software Gen5 (Biotek, Germany) and confirmed on a 1.7% (w/v) agarose gel.

### High-throughput sequencing and reference assembly

Genome sequences were obtained individually from each individual using 1 µg of DNA, on a Genome Sequencer Illumina HiSeq 4000 technology (125 bp PE; Fig. 1). A total of 539,473,738 reads were generated on both sequencings. Low quality reads (phred quality lower than 15) and reads without mate were removed. Additionally, low quality bases were removed from 3′ and 5′ ends. Minimum read size obtained after base trimming was 36. For the WF genome, 216,895,404 reads (91.6% of the total) were used on further analyses, while the WM genome was represented with 277,949,176 high quality reads (91.8%). Considering the *V. v. vinifera* cv. PN40024 reference genome available^1^, we estimated a genome size of about 486 Mbp, for which our predicted coverage ranges between 56X (WF) and 72X (WM). For general proposes, the results obtained from the different individuals were merged together, to better represent a wild- type genome.

The good-quality reads were mapped against the reference genome^1^ using BWA (0.7.15)^45^ with the default parameters. About 97% of the input reads mapped correctly to the reference genome (Table 1).

Considering the artificial coverage created due to PCR amplification during library preparation, the reads were submitted to Picard (1.131; https://broadinstitute.github.io/picard/). The MarkDuplicates tool from Picard was used to detect duplicated reads whose origin is expected to be a single fragment of DNA. MarkDuplicates marks the reads as duplicates if multiple reads pairs maps in the same genomic location and with the same orientation. The presence of insertions and deletions, on the new obtained genomes, compared to the reference genome, promoted the occurrence of punctual misalignments. These misalignments were locally realigned with GATK (3.5)^46,47^. Taking into consideration the reported quality score, sequencing cycle and context, a final Base Quality Recalibration was performed with GATK, to improve the base quality score of the reads. About 92% of the mapped reads were kept after these three refinement steps. Whenever not explicitly indicated, default parameters were used.

### Variant analyses

SNP and InDel identification between the wild genomes and the reference genome, was performed with GATK’s Haplotype Caller^46,47^ and annotation according to the gene context was achieved with snpEff (4.3; https://snpeff.sourceforge.net), according to PN40024 12X V2.1. The snpEff defaults for upstream and downstream were kept (5 kbp) and, when upstream and downstream regions overlap (adjacent genes), we considered the most relevant context: upstream instead of downstream. Variant quality was estimated with GATK’s Variant Annotator module^46,47^ and false positives were filtered with GATK’s Variant Filtration module^46,47^. Options for GATK’s Variant Filtration module were: (1) LowCovFilter: ≤ 20; (2) QDFilter: < 2.0; (3) MQFilter: < ** − **12.5; (4) FSFilter: > 60.0 (> 200.0 of InDels); (5) HaplotypeFilter: > 13.0; (6) MQFilter: < ** − **12.5 and (7) ReadPosFilter: < ** − **8.0 (< **− **20.0 for InDels). Analysis were performed on RStudio (1.0.143)^48^, running over R (3.4.3)^49^. Outliers were estimated based on Tukey's fences^50^, considering k = 1.5. Venn Diagrams were obtained from calculate.overlap and draw.pairwise.venn functions (VennDiagram package, version 1.6.17^51^). Heatmap analysis on SNPs and InDels density along chromosomes was performed with the R build-in heatmap function.

Transitions over transversion ratio (Ti/Tv) as well as heterozygous over non-reference homozygous SNPs (he/nonref-hom) were calculated for each genome with SNPs grouped by chromosome (discarding the “Un” and “X_random” virtual chromosomes). Calculations were performed with Python (3.7.4) running over Jupyter Notebook (6.0.1). Modules pandas (0.25.1) and matplotlib (3.1.1) were used to manipulate data and draw boxplots. Whenever not explicitly indicated, default parameters were used. Data from WF and WM was treated separately and pooled only for plotting and conclusions.

### De novo assembly

A de novo assembly of the genomes was performed in order to obtain the genomic sequences of *loci* that were unclear on the reference genome. From the bulk of sequenced reads (Table 2), an assessment was performed using FastqC software (v0.11.7). Considering the quality indicators, the reads were cleaned with BBDuk (v38.01) from BBMap package. A pipeline was set to remove adapters from both ends (ktrim = r and ktrim = lm; with options k = 23, tpe and tbo, using the provided adapters.fa as reference) and low quality reads (options qtrim = rl and trimq = 20). Reads were prepared for assembly using fq2fa function from IDBA package (1.1.3)^52,53^, with options –merge and –filter. Assembly was conducted with idba_ud. The contigs obtained with less than 1,000 bp were removed, as this represent low informative segments.

Contigs obtained on de novo assemblies were queried against the genes annotated on the sex region^15,16^, retrieved from the reference genome (hermaphrodite), on a blastn^54,55^ run with e-value set to 1e − 10. The selected contigs were manually inspected. Although the usage of only two individuals, one of each sex, lacks the resolution to differentiate between sex-specific patterns and individual evolutionary mutations, this approach may provide a little insight into the genetic differences leading to different flower types.

The results of this assembly were validated by confirming the presence of contigs associated with *VviAPRT3* and *VviFSEX* as reported by Coito et al.^17^ and *VviINP1*^18,19^, *VviPLATZ*^18^ and *VviYABBY*^19^ and by testing the heterozygosity overlap between both assemblies (Supplementary Table S1). We manually inspected the region of chromosome 2 that has been associated with sex^15,16^. Whenever not explicitly indicated, default parameters were used.

### RNA editing

The SNPs data obtained here was compared against the transcriptomic data obtained for the same individuals in 2014^32^. RNA was extracted from inflorescences WF and WM individuals at developmental stages B, D and H^56^. Total RNA was extracted with Spectrum Total RNA kit (Sigma-Aldrich, Spain), according to the manufacturer’s instructions. cDNA libraries were obtained along standard Illumina pipeline, targeting poly-A tails. The obtained transcriptomic sequences were assembled against the reference genome (PN40024–12X), in transcriptome version 1^2^. SNPs were assessed between the transcriptomes and reference genome with CLC Genomics Workbench (Quiagen). Calls were discarded if: (1) the SNP call was within the first or last 60 bases of a linkage group sequence; (2) the reference genome base was ambiguous at the SNP position or within 60 bases of it; (3) the average copy number of the SNP flanking region was more than two; (4) the Illumina quality score of either allele was less than 20 and (5) the number of reads supporting either allele was less than two reads per accession. The initial list of variants was filtered using the Phred quality scores of the position and surrounding bases. To reduce false positives in variant calling, the minimum variant frequency was set to 15%. The minimum coverage required was 4 reads. Gene expression was measured using the number of Reads per Kilobase of exon model per Million mapped reads (RPKM).

Comparison between the RNA-seq data and the genomes sequenced was performed on R (3.4.3)^49^, running on a RStudio cluster (1.0.143)^48^. The comparison between the genome and the transcriptome was performed by assessing the exact genomic location of the SNPs, as both studies were mapped against the same reference genome. To guarantee low number of false positives, we have defined a set of conserved filters to increase confidence: (1) Each analysed site should have a minimum coverage of 5X (both in DNA-seq and RNA-seq, simultaneously); (2) the gene should be expressing in all samples (WF and WM individuals at inflorescence developmental stages B, D and H; RPKM ≥ 1); (3) events as deletions and inserts have been removed and (4) at least 10% of the reads covering the region should identify the nucleotide, otherwise that nucleotide was considered an error and discarded.

For sites where a SNP was identified only in DNA-seq or RNA-seq, a deeper analysis was performed, by assessing the information in the BAM files. In these situations, we evaluated if the lack of information was based on similarity between the genome/transcriptome^32,33^ sequenced, and the reference genome, or if the position was discarded by the filters defined. To the remaining sites, the homozygosity of the locus was computed considered the frequency of each allele obtained from the genome sequence described in this paper (freq ≥ 0.90, homozygous; freq < 0.90, heterozygous). The comparison between the genome observed nucleotides and the transcriptome was achieved and, when the nucleotides observed in the transcriptome were not supported by the genomic information, we have considered RNA editing sites. We note that false RNA editing positives may be identified where the nucleotide observed in the transcriptome is present in less than 9% of the genome reads.

Interesting genes (reported in Fig. 6) were manually inspected using the Integrated Genome Viewer (IGV) browser^57^, loaded with the reference genome (PN40024–12X), the most recent gene annotation (V2.1) and the BAM files corresponding to the genome and transcriptomes sequencings, to validate the overall R analysis, with success.

As RNA editing was assessed based on cDNA libraries, we decided to refer to the DNA–RNA differences using the DNA and cDNA nomenclature (for example, using Thymine instead of Uracyl).

The R scripts developed will be available upon request, due to script’s authors stringencies.

### RNA editing validation by Sanger sequencing

For individual WM and a secondary female individual (WF_2), RNA and DNA were extracted from the same inflorescence sample, at different developmental stages using Spectrum Plant Total RNA Kit (Sigma-Aldrich) and DNeasy Plant Mini Kit (Qiagen, Valencia, CA, USA), respectively, following manufacturer’s instructions. This approach allows to exclude putative somatic mutations that may occur along the development of the new branches. Total RNA was additionally treated with 30 units of DNase I (Qiagen). Nucleic acid concentration was measured using a microplate reader Synergy HT (Biotek, Germany), with the software Gen5 (Biotek, Germany). The cDNA was synthesized from 100 ng of total RNA, using oligo(dT)_20_ and RevertAid Reverse Transcriptase (Thermo Fisher Scientific) in a 20 μL reaction volume, following the manufacturer’s instructions. cDNA concentration was measured as described above.

Two genes in chromosome 2, *VviYABBY* (VIT_202s0154g00070) and VIT_202s0033g01400 were manually inspected using IGV. Primers were designed for exons of *VviYABBY* and VIT_202s0033g01400 genes using Primer Premier5 (Supplementary Table S2). For *VviYABBY*, a 336 bp fragment (DNA and cDNA) was amplified and for VIT_202s0033g01400 a 829 bp fragment was obtain in DNA and 632 bp in cDNA amplification.

The DNA and the cDNA amplifications were performed through PCR in 25 μL total volume composed by 0.2 µg of DNA (or cDNA), PCR buffer (20 mM Tris–HCl [pH 8.4], 50 mM KCl), 1.5 mM of Mg_2_, 0.2 mM of dNTP mix, 0.4 μM of each forward and reverse primers, 10 U of Taq DNA polymerase and autoclaved MiliQ water. The applied program had 3 min for initial denaturation at 95 °C followed by 34 cycles of 45 s at 95 °C (denaturation), 45 s at 50 °C (annealing) for *VviYABBY* and 55 °C for VIT_202s0033g01400, and 90 s at 72 °C (extension), followed by a final extension for 5 min at 72 °C. PCR amplifications (1 μL) were inspected on an agarose gel (1%). The remaining product was purified using PureLink Quick PCR Purification kit (Thermo Fisher Scientific), according to the kit protocol. For each gene DNA and cDNA were Sanger sequenced in three technical replicates without cloning. The provided electropherograms were manually inspected.

### Variant detection validation

Four genes involved in flowering development were selected and specific primers were designed (Supplementary Table S2) using Primer Premier 5 (Premier Biosoft). Amplification reactions were performed in 25 µL composed by1 µg of DNA, PCR buffer (20 mM Tris–HCl [pH 8.4], 50 mM KCl), 1.5 mM of MgCl_2_, 0.2 mM of dNTP mix, 0.4 μM of each forward and reverse primers, 5 U of Taq DNA polymerase and autoclaved MiliQ water. The applied program had 4 min for initial denaturation at 94 °C; 30 cycles at 94 °C 45 s (denaturation), annealing at specific temperature (Supplementary Table S2) 45 s (annealing) and 72 °C 90 s (extension); and a final extension at 72 °C for 4 min. PCR products were purified using PureLink (Invitrogen) and sequenced by Sanger without cloning.

## Supplementary information

Supplementary Information 1.

Supplementary Information 2.

Supplementary Information 3.

## Data Availability

The raw data analysed in this study was submitted to the Sequence Read Archive (SRA) database (www.ncbi.nlm.nih.gov/sra/) under the number PRJNA549602. Scripts are available at the GitHub repository https://github.com/sex-group/wild-grapevine-genome. The transcriptomes used in the RNA editing analysis are available at the Gene Expression Omnibus (GEO) database (https://www.ncbi.nlm.nih.gov/geo/) with the accession GSE56844.
