# Supplementary material for: Portuguese wild grapevine genome re-sequencing (Vitis vinifera sylvestris)
Source: Sci Rep. 2020 Nov 4;10:18993. doi: 10.1038/s41598-020-76012-6 (PMC7642406; doi:10.1038/s41598-020-76012-6)
Supplement: Supplementary file 1 — Supplementary Information 1. [file 41598_2020_76012_MOESM1_ESM.pdf]

Portuguese wild grapevine genome re-sequencing (*Vitis vinifera sylvestris*)

Miguel J N Ramos[1*], João L Coito[1], David Faísca-Silva[1], Jorge Cunha[2], M Manuela R Costa[3], Sara Amâncio[1], Margarida Rocheta[1*]

[1] LEAF, Linking Landscape, Environment, Agriculture and Food, Instituto Superior de Agronomia, Universidade de Lisboa, Tapada da Ajuda 1349-017 Lisboa, Portugal
[2] Instituto Nacional de Investigação Agrária e Veterinária, Quinta d'Almoinha, 2565-191 Dois Portos, Portugal
[3] Plant Functional Biology Centre, Biosystems and Integrative Sciences Institute, University of Minho, 4710-057 Braga, Portugal

Corresponding authors
* mramos@isa.ulisboa.pt
* rocheta@isa.ulisboa.pt

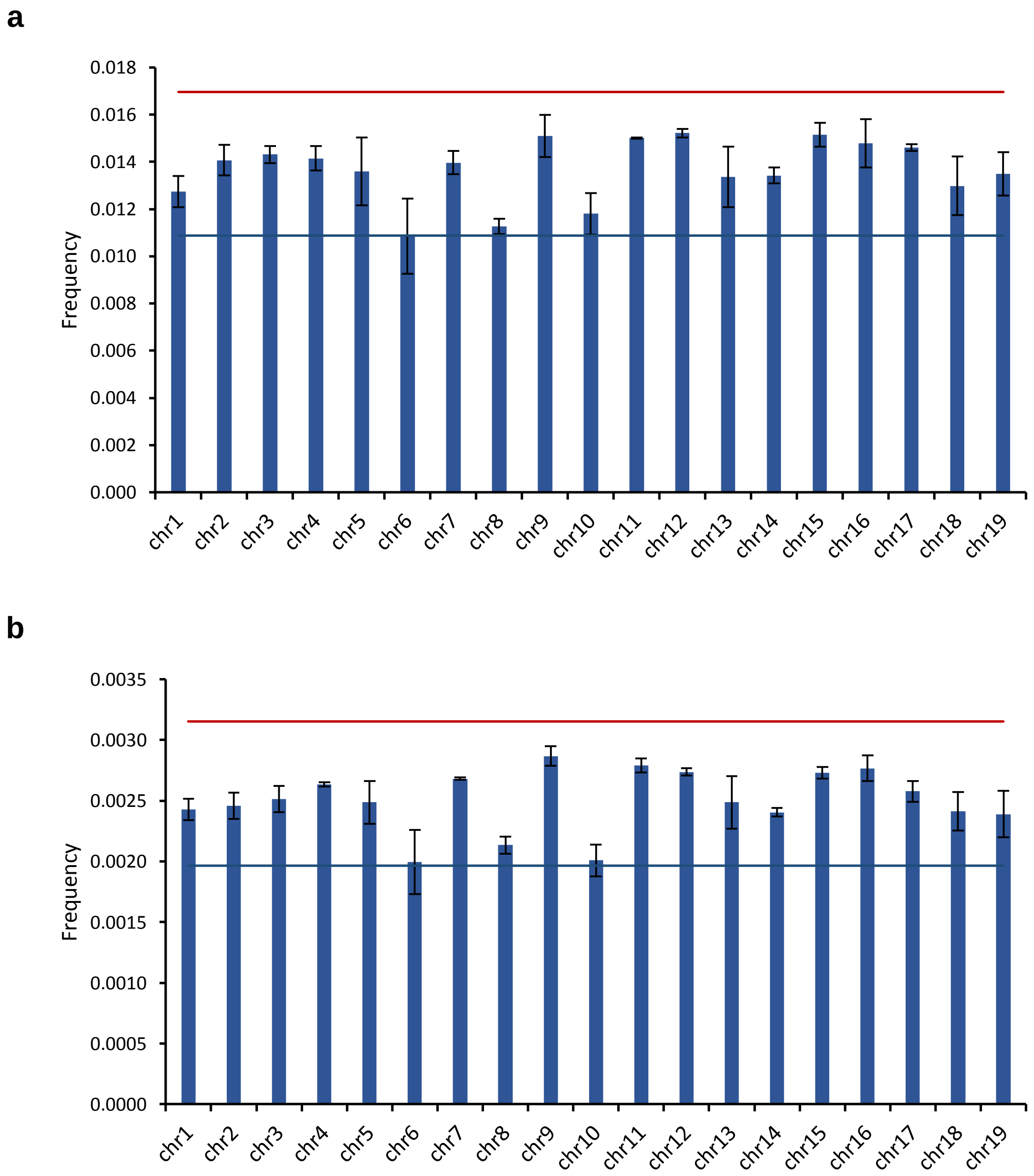

Supplementary Figure S1. Frequency of events per chromosome length (base pairs). (a) SNPs. (b) InDels. Red and blue lines represent the Tukey's outlier fences (k = 1.5). Vertical bars represent the standard error.

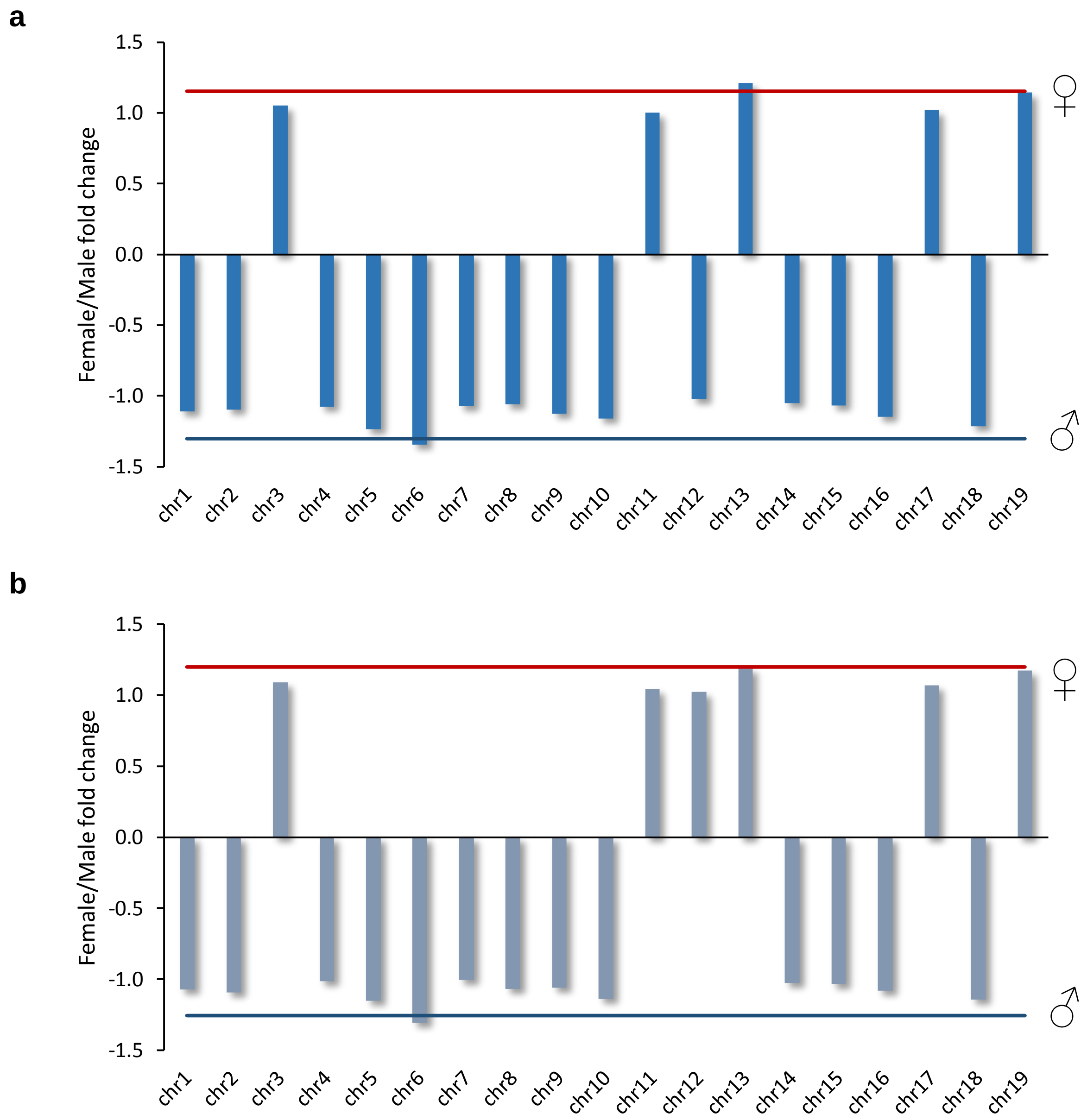

Supplementary Figure S2. Fold change variation (occurrences in WF/WM).
(a) SNPs. (b) InDels. Red and blue lines represent the Tukey's outlier fences (k = 1.5).

a

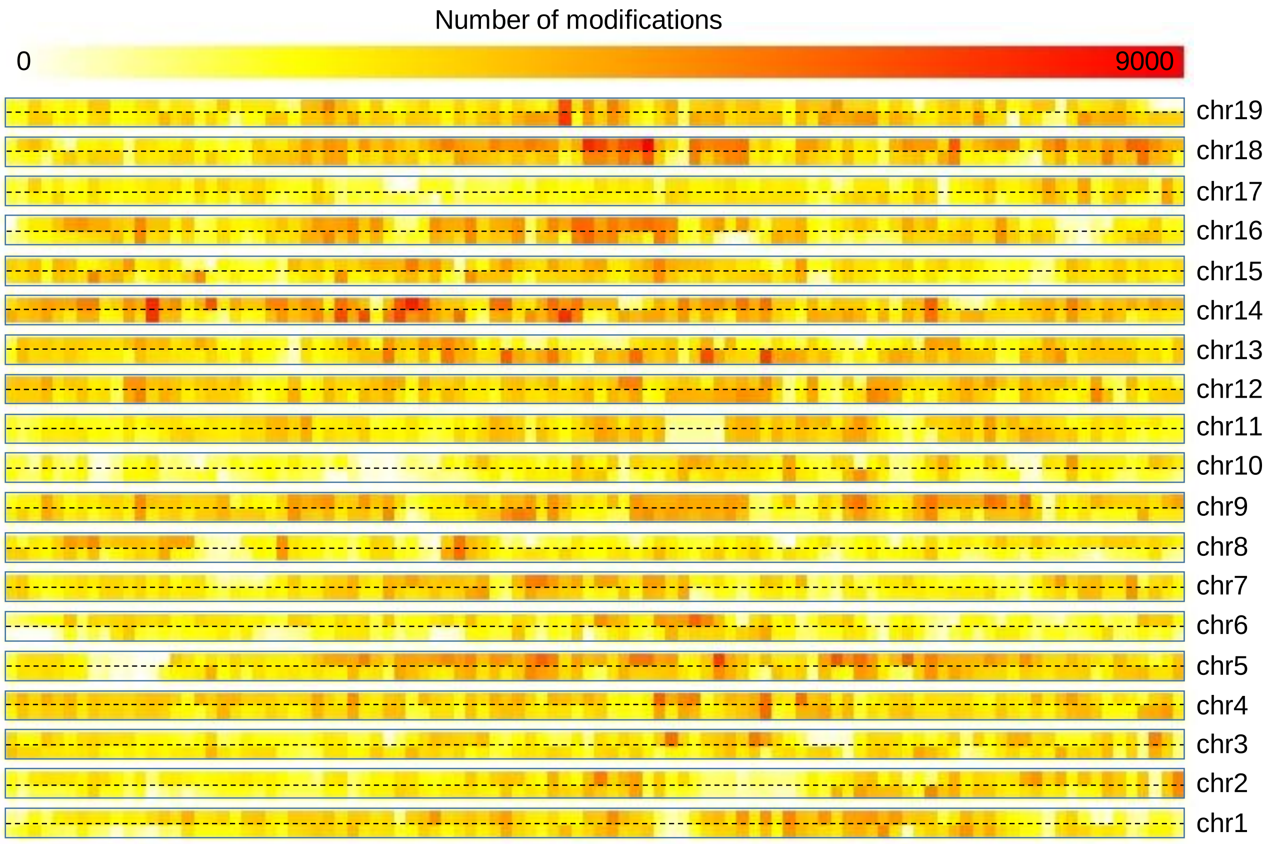
Number of modifications
0
9000
chr19
chr18
chr17
chr16
chr15
chr14
chr13
chr12
chr11
chr10
chr9
chr8
chr7
chr6
chr5
chr4
chr3
chr2
chr1

b

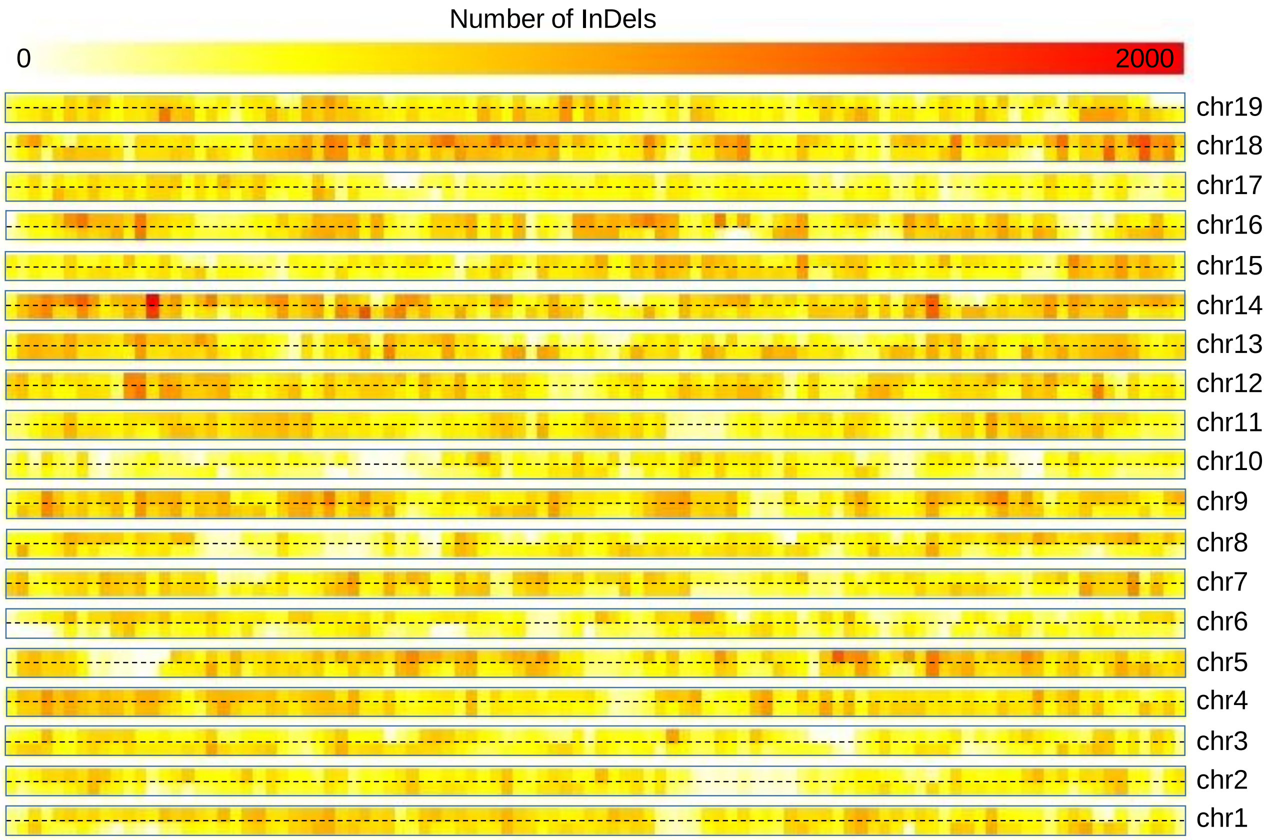
Number of InDels
0
2000
chr19
chr18
chr17
chr16
chr15
chr14
chr13
chr12
chr11
chr10
chr9
chr8
chr7
chr6
chr5
chr4
chr3
chr2
chr1

Supplementary Figure S3. Heatmap density representing the number of SNPs (a) and indels (b) identified for each $1/100^{th}$ of the chromosomes (the virtual X_random and Unknown chromosomes were not considered). For each chromosome, the upper and lower row represents the WM and WF individual, respectively. Both compared against the reference genome, PN40024, CRIBI, 12X. Colour gradation represent the density of modification.

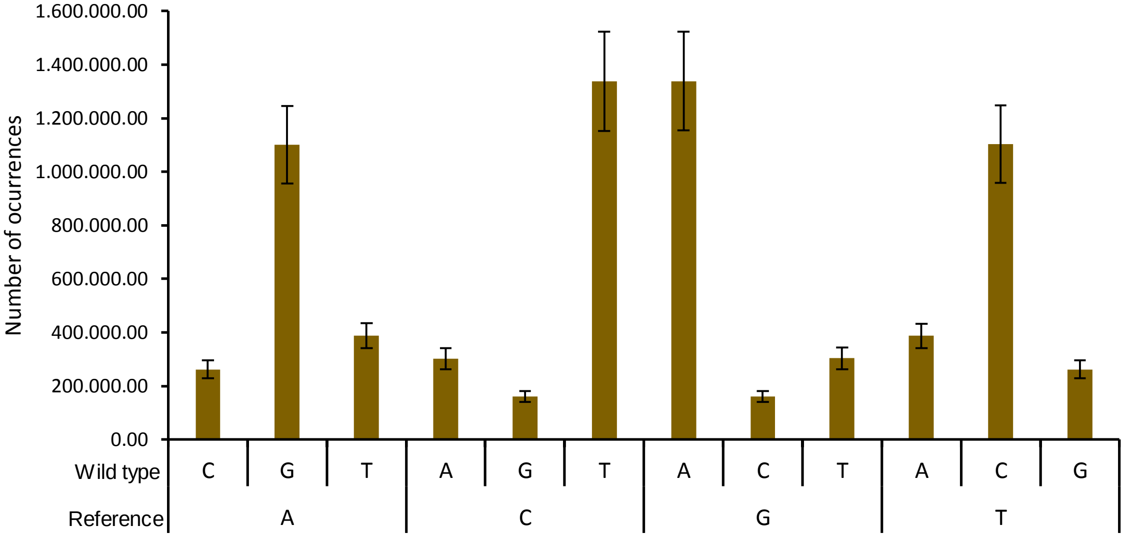

Supplementary Figure S4. Number of transversions (A ↔ C, A ↔ T, C ↔ G and G ↔ T) and transitions (A ↔ G and C ↔ T). Wild type: the nucleotides identified in the wild-type individuals; Reference: The nucleotide in the reference genome (PN40024, CRIBI, 12X). Vertical bars represent the standard error.

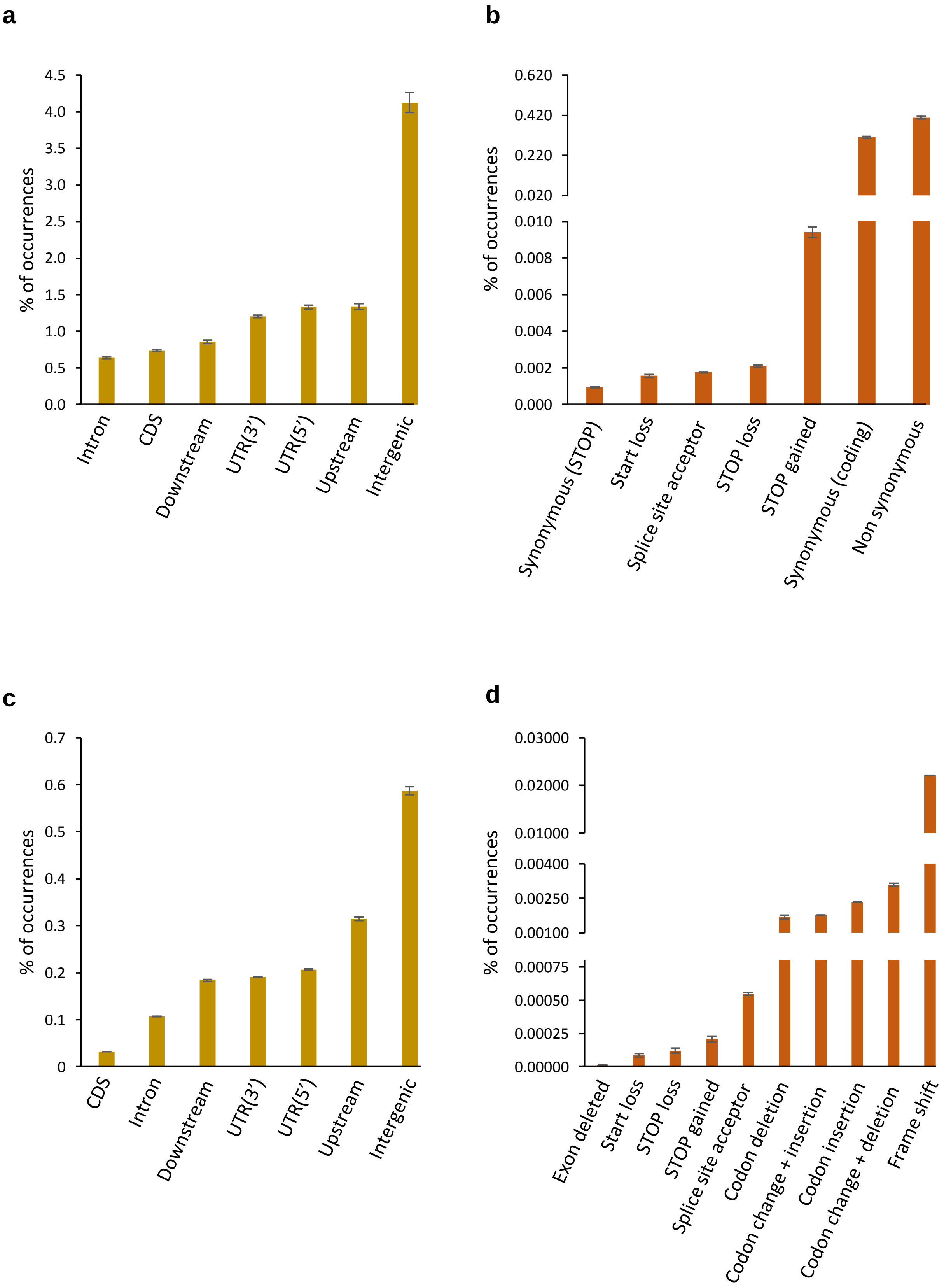

Supplementary Figure S5. The gene context of the identified modification in wild-type genomes. (a) Percentage of regions affected by SNPs and (b) the impact caused by the SNP when it occurs in CDS regions. (c) Percentage of features affected by InDels and (d) its impact in CDS regions. Reference genome and annotation: PN40024, CRIBI, 12X V2.1. For events between genes, the most relevant context was chosen (e.g. upstream instead of downstream), as described by SnpEff documentation. Upstream and downstream were considered for a 5 kbp range. Vertical bars represent the standard error.

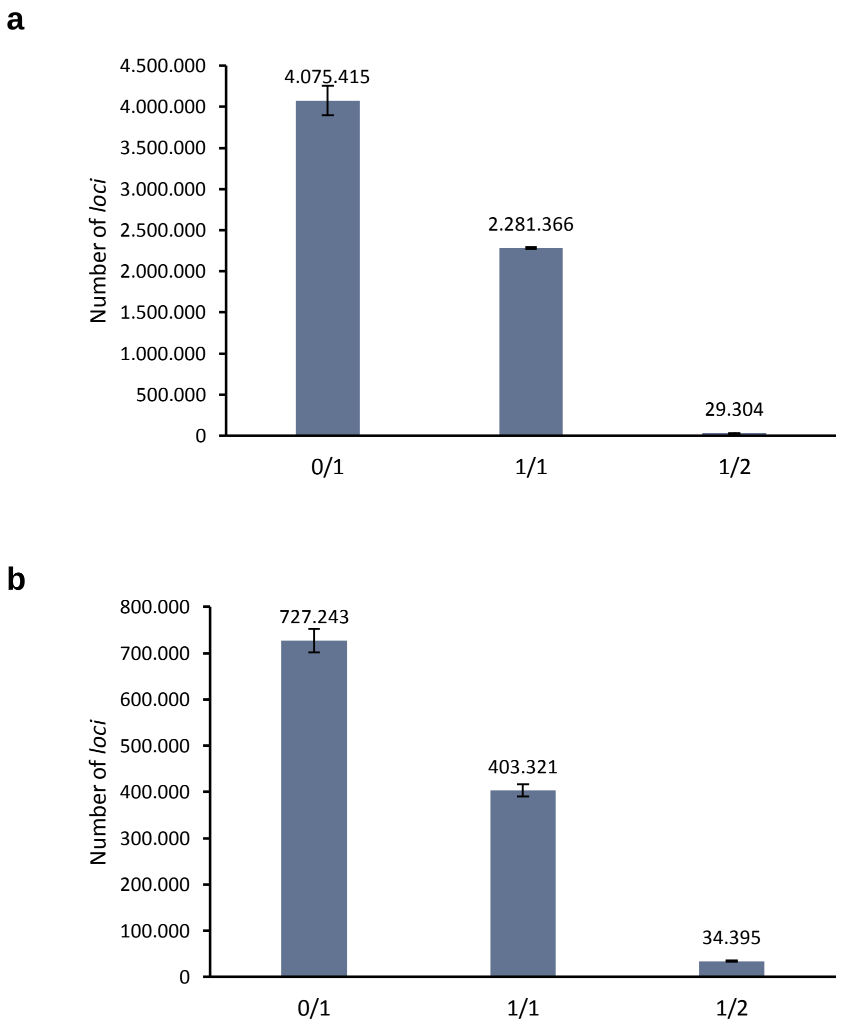

Supplementary Figure S6: Number of homozygous (1/1) and heterozygous (0/1 and 1/2) *loci* in *V. v. sylvestris* genomes. (a) Evaluation according to SNPs. (b) Evaluation according to InDels. 0/1 represents *loci* where two alleles were observed, one being identical to reference genome (PN40024, CRIBI, 12X); 1/1 represent *loci* where only one allele was observed, being different than reference; 1/2 represents heterozygous *loci*, being both alleles different than reference. Vertical bars represent the standard error.

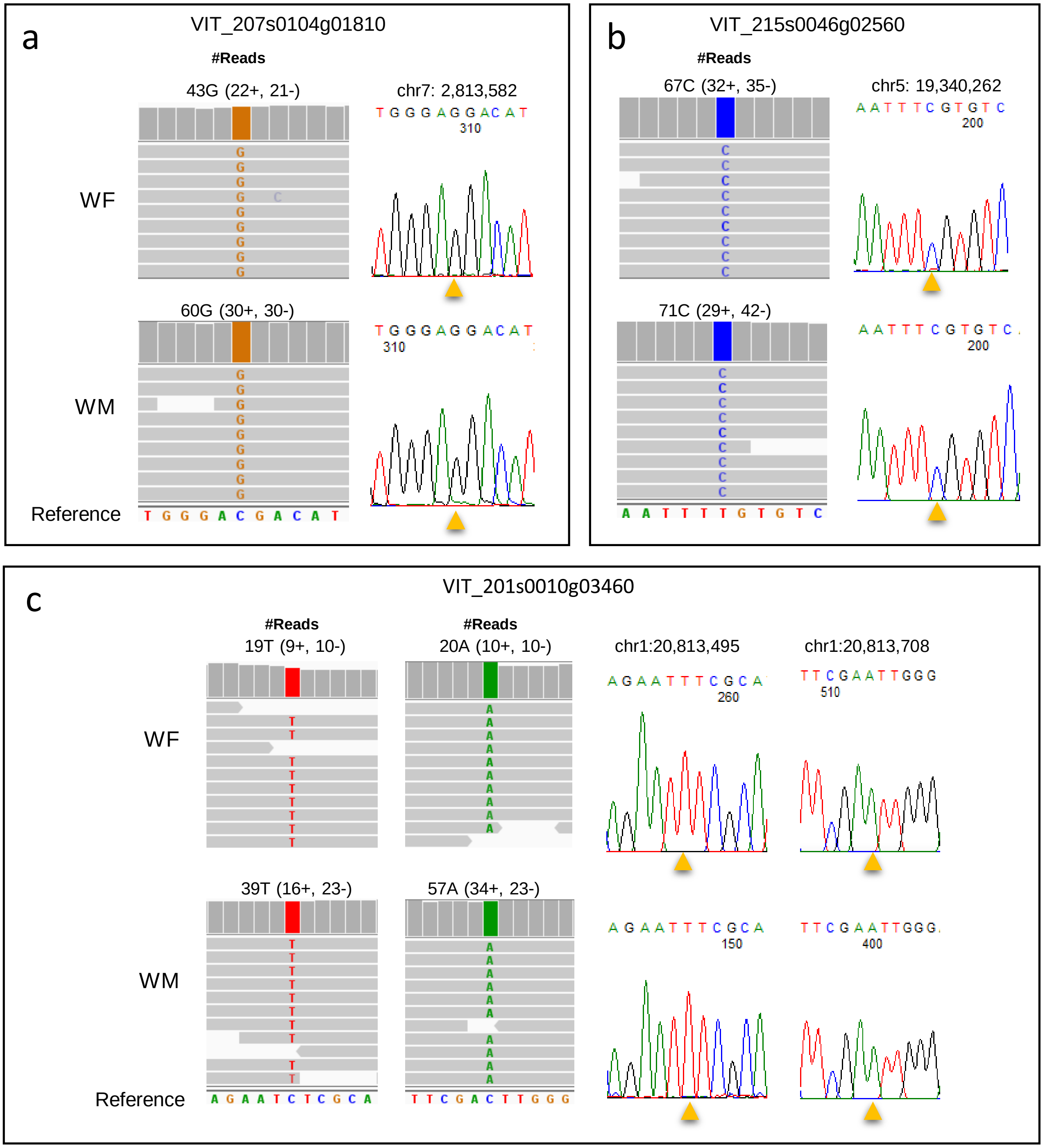
a
VIT_207s0104g01810
#Reads
43G (22+, 21-)
chr7: 2,813,582
T G G G A G G A C A T
310
WF
60G (30+, 30-)
T G G G A G G A C A T
310
WM
Reference
T G G G A C G A C A T
b
VIT_215s0046g02560
#Reads
67C (32+, 35-)
chr5: 19,340,262
A A T T T C G T G T C
200
71C (29+, 42-)
A A T T T C G T G T C
200
A A T T T T G T G T C
c
VIT_201s0010g03460
#Reads
19T (9+, 10-)
#Reads
20A (10+, 10-)
chr1:20,813,495
chr1:20,813,708
A G A A T T T C G C A
260
T T C G A A T T G G G
510
WF
39T (16+, 23-)
57A (34+, 23-)
A G A A T T T C G C A
150
T T C G A A T T G G G
400
WM
Reference
A G A A T C T C G C A
T T C G A C T T G G G

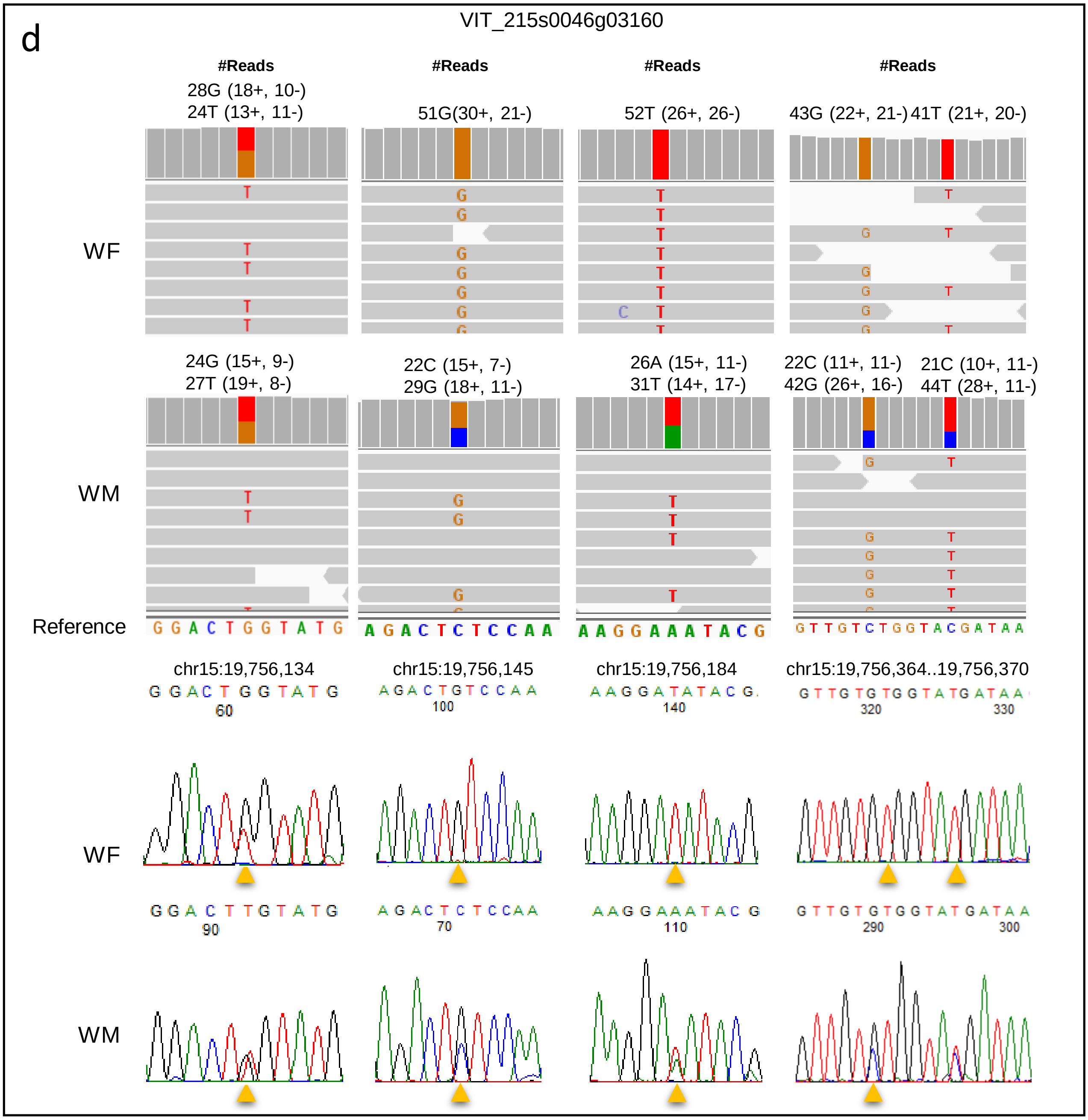

Supplementary Figure S7. Validation on wild-type identified modifications in both studied individuals. (a) Amplification of *VviATGSTF13* (VIT_207s0104g01810) representing a homozygous modification on chr7:2,813,582, shared by both individuals. (b) Amplification of *VviGL3* (VIT_215s0046g02560) where a homozygous change is identified on both individuals (chr5:19,340,262). (c) Amplification of *caffeoyl-o-methyltransferase* (VIT_201s0010g03460) with homozygous and shared events on both wild-type individuals (chr1:20,813,495..20,813,708). (d) Amplification of an *adenosine deaminase* (VIT_215s0046g03160), where substitutions are heterozygous and shared between individuals (chr15:19,756,134) and distinct between them (chr15:19,756,145.. 19,756,370). On all panels, the first representation, was obtained by Integrated Genome Viewer (IGV) and represents the substitution observed in genome sequencing. For each substitution the number of reads covering the position on each sequencing direction is indicated. The electropherograms show evidence of nucleotide modification when compared with the reference. Orange arrows, indicate the substitution event. Reference genome, PN40024, CRIBI, 12X.

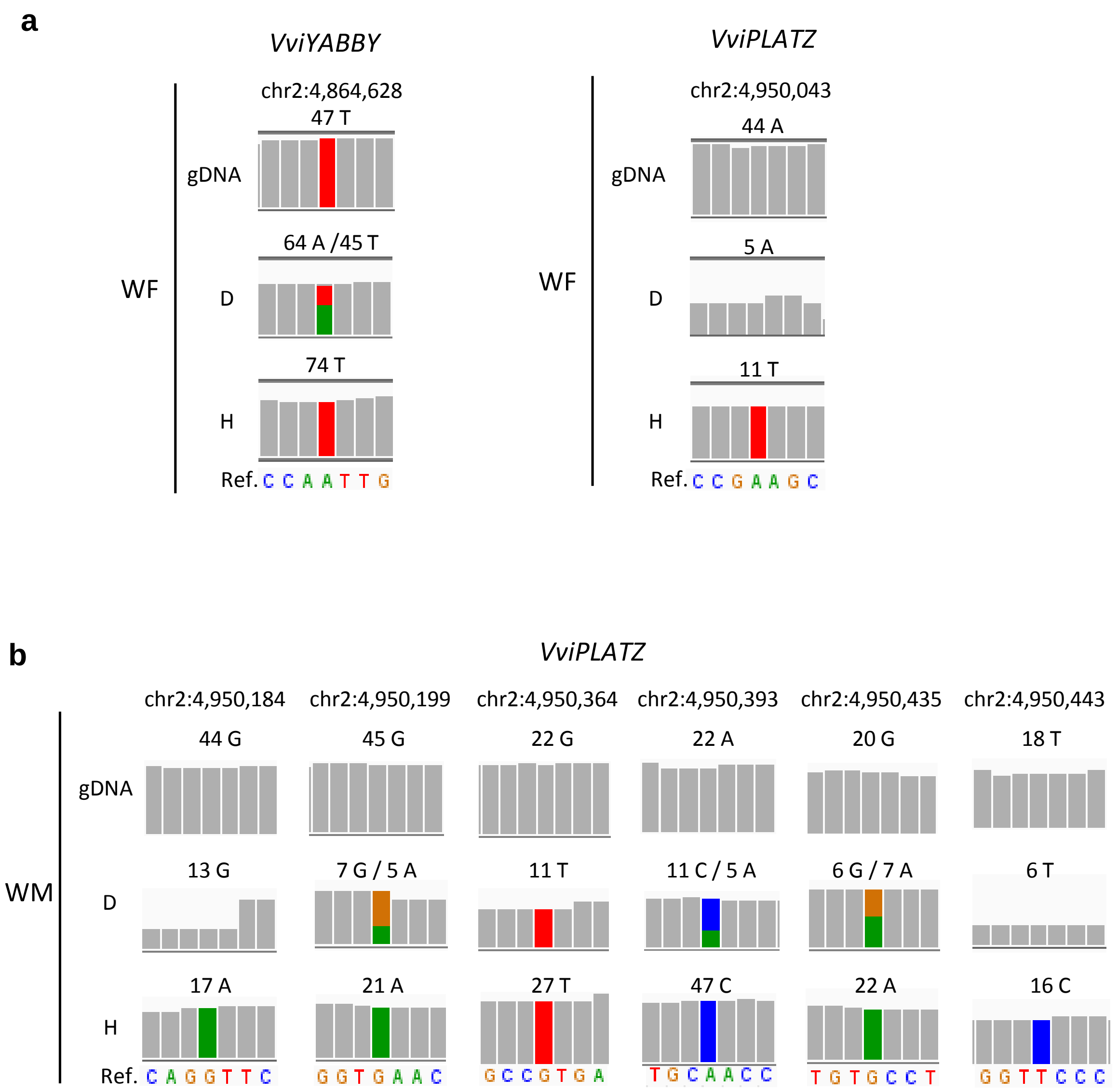

Supplementary Figure S8. Visualization through Integrated Genome Viewer (IGV) of *VviYABBY* and *VviPLATZ* in developmental stages D and H. (a) In WF plant *VviYABBY* show modification in both stages but *VviPLATZ* only present editing at stage H. (b) WM present some modifications in both stages. For each editing event the number of reads covering the position is indicated. Ref, Reference genome, PN40024, CRIBI, 12X; gDNA, genomic DNA; D and H, developmental stages; chr2, chromosome 2, editing position.

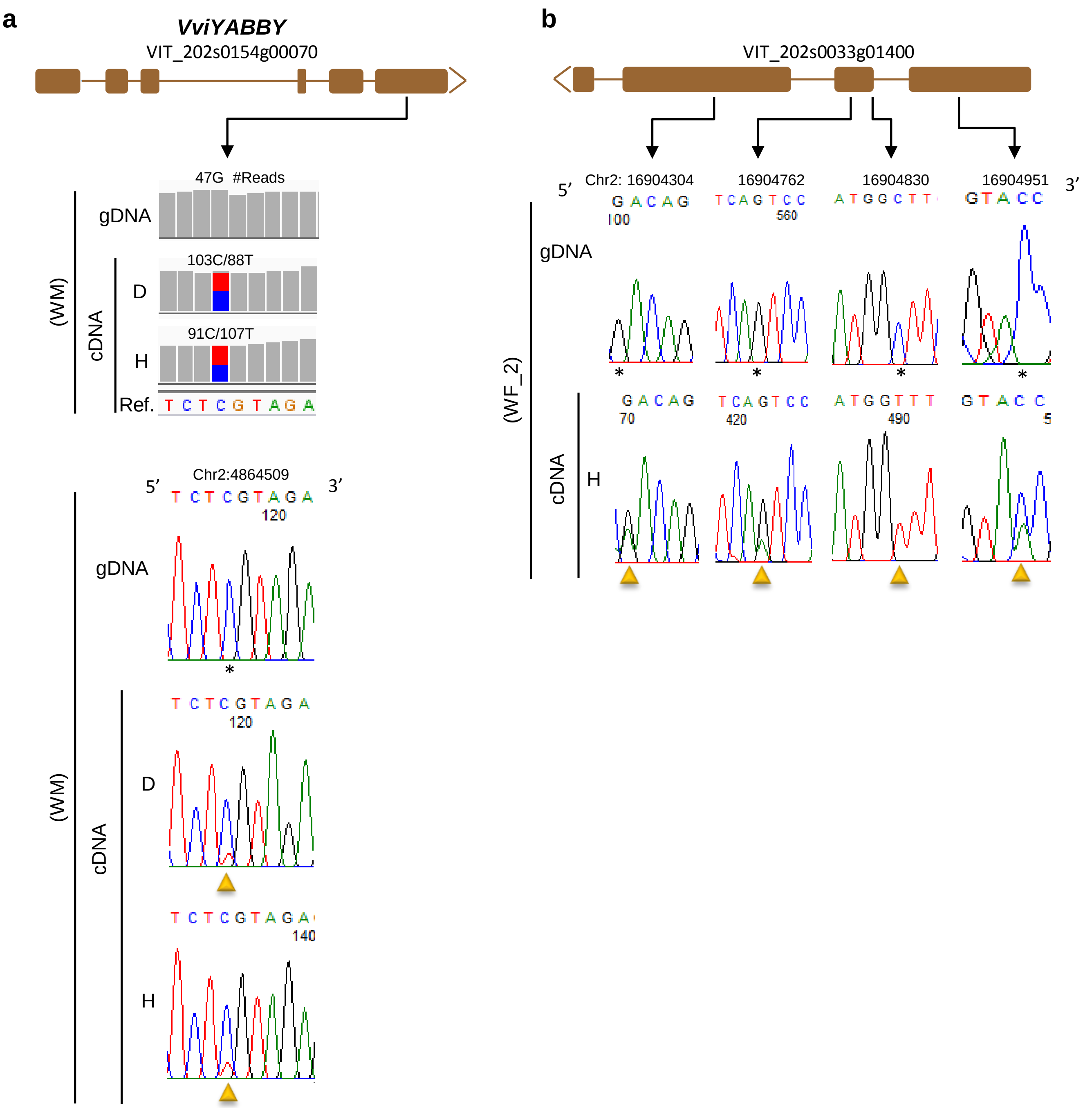

Supplementary Figure S9. RNA editing events in *VviYABBY* and VIT_202s0033g01400 genes.
a) mRNA modifications in the *VviYABBY* (VIT_202s0154g00070) gene in male inflorescences. We show *VviYABBY* gene structure where brown bars represent exons and line introns. Through the visualization of DNA-seq and RNA-seq, one RNA editing event within the *VviYABBY* are observed. Results were validated through Sanger sequencing, in the male plant (WM) used in transcriptomic data. Electropherograms confirm the mRNA two-variants at the development stages D and H. (b) RNA editing events in the VIT_202s0033g01400 gene in a different female plant (WF_2) at developmental stage H. In the gene structure brown bars represent exons and line introns. The Sanger sequencing of this gene section revealed three events where edited and unedited mRNA are observed and one event with a nucleotide change C-to-T.
