# Supplementary material for: Portuguese wild grapevine genome re-sequencing (Vitis vinifera sylvestris)
Source: Sci Rep. 2020 Nov 4;10:18993. doi: 10.1038/s41598-020-76012-6 (PMC7642406; doi:10.1038/s41598-020-76012-6)
Supplement: Supplementary file 2 — Supplementary Information 2. [file 41598_2020_76012_MOESM2_ESM.pdf]

Portuguese wild grapevine genome re-sequencing (*Vitis vinifera sylvestris*)

Miguel J N Ramos[1*], João L Coito[1], David Faísca-Silva[1], Jorge Cunha[2], M Manuela R Costa[3], Sara Amâncio[1], Margarida Rocheta[1*]

[1] LEAF, Linking Landscape, Environment, Agriculture and Food, Instituto Superior de Agronomia, Universidade de Lisboa, Tapada da Ajuda 1349-017 Lisboa, Portugal

[2] Instituto Nacional de Investigação Agrária e Veterinária, Quinta d'Almoinha, 2565-191 Dois Portos, Portugal

[3] Plant Functional Biology Centre, Biosystems and Integrative Sciences Institute, University of Minho, 4710-057 Braga, Portugal

Corresponding authors

* mramos@isa.ulisboa.pt

* rocheta@isa.ulisboa.pt

Supplementary Table S1. Detailed information of gene in chromosome 2 (for details, see Figure 6) and comparation to the reference assembly. Contigs retrieved: Number of contigs obtained after *de novo* assembly. Alleles: Number of alleles identified in *de novo* and reference assemblies. NA represents genes to which was not possible to retrieve information, as contigs were too fragmented.

**Accession**	**Contigs retrieved**		**Alleles** (*de novo* assembly)		**Alleles** (reference assembly)	
	**Female**	**Male**	**Female**	**Male**	**Female**	**Male**
VIT_202s0234g00050	NA	NA	NA	NA	2	2
VIT_202s0025g00140	1	1	1	1	1	1
VIT_202s0025g00200	1	2	1	2	1	2
VIT_202s0025g00530	4	4	1	1	1	2
VIT_202s0025g01620	4	1	2	1	2	1
VIT_202s0025g01630	1	3	1	2	2	1
VIT_202s0025g01710	2	2	2	2	2	2
VIT_202s0025g01730	1	4	1	2	2	2
VIT_202s0025g02630	1	1	1	1	1	1
VIT_202s0025g02690	1	6	1	2	1	1
VIT_202s0025g03000	1	1	1	1	1	1
VIT_202s0025g04390	2	13	2	2	2	2
VIT_202s0241g00140	1	1	1	1	1	1
VIT_202s0241g00150	NA	NA	NA	NA	1	1
VIT_202s0241g00160	NA	NA	NA	NA	2	2
VIT_202s0241g00170	1	1	1	1	1	1
VIT_202s0012g00660	1	1	1	1	1	1
VIT_202s0012g01270	7	6	2	2	2	2
VIT_202s0012g02060	1	1	1	1	1	1
VIT_202s0033g00020	2	1	2	1	2	1
VIT_202s0033g01380	1	6	1	2	1	2
VIT_202s0033g01400	1	1	1	1	1	1
VIT_202s0154g00010	1	1	1	1	1	1
VIT_202s0154g00020	9	12	2	2	2	2
VIT_202s0154g00030	1	1	1	1	1	1
VIT_202s0154g00040	1	9	1	2	2	2
VIT_202s0154g00050	1	3	1	2	1	1
VIT_202s0154g00060	1	1	1	1	2	2

Accession	Contigs retrieved		Alleles (*de novo* assembly)		Alleles (reference assembly)	
	**Female**	**Male**	**Female**	**Male**	**Female**	**Male**
VIT_202s0154g00070	1	9	1	2	1	2
VIT_202s0154g00080	2	21	2	2	2	2
VIT_202s0154g00090	1	5	1	1	1	2
VIT_202s0154g00100	NA	NA	NA	NA	2	2
VIT_202s0154g00110	1	NA	1	NA	2	1
VIT_202s0154g00120	1	2	1	2	1	1
VIT_202s0154g00130	1	2	1	2	1	1
VIT_202s0154g00140	1	27	1	2	2	1
VIT_202s0154g00150	1	10	1	2	1	1
VIT_202s0154g00160	1	8	1	2	2	1
VIT_202s0154g00170	1	1	1	1	2	2
VIT_202s0154g00180	1	9	1	2	2	1
VIT_202s0154g00190	1	14	1	1	2	1
VIT_202s0154g00200	2	12	2	2	2	1
VIT_202s0154g00210	1	2	1	2	1	2
VIT_202s0154g00220	1	2	1	2	1	1
VIT_202s0154g00230	1	2	1	1	1	1
VIT_202s0154g00240	2	10	2	2	2	2
