# Supplementary material for: Portuguese wild grapevine genome re-sequencing (Vitis vinifera sylvestris)
Source: Sci Rep. 2020 Nov 4;10:18993. doi: 10.1038/s41598-020-76012-6 (PMC7642406; doi:10.1038/s41598-020-76012-6)
Supplement: Supplementary file 3 — Supplementary Information 3. [file 41598_2020_76012_MOESM3_ESM.pdf]

Portuguese wild grapevine genome re-sequencing (*Vitis vinifera sylvestris*)

Miguel J N Ramos[1*], João L Coito[1], David Faísca-Silva[1], Jorge Cunha[2], M Manuela R Costa[3], Sara Amâncio[1], Margarida Rocheta[1*]

[1] LEAF, Linking Landscape, Environment, Agriculture and Food, Instituto Superior de Agronomia, Universidade de Lisboa, Tapada da Ajuda 1349-017 Lisboa, Portugal

[2] Instituto Nacional de Investigação Agrária e Veterinária, Quinta d'Almoinha, 2565-191 Dois Portos, Portugal

[3] Plant Functional Biology Centre, Biosystems and Integrative Sciences Institute, University of Minho, 4710-057 Braga, Portugal

Corresponding authors

* mramos@isa.ulisboa.pt

* rocheta@isa.ulisboa.pt

Supplementary Table S2. Primers designed to validate the contigs obtained under *de novo* assembly and to validate RNA editing, through a Sanger analysis. For each amplified gene the forward and reverse primers (in 5' to 3' orientation) are provided, as well as, the annealing temperature used for PCR amplification.

Gene ID	Orientation	Primer sequence (5' >> 3')	Tm (ºC)
VIT_201s0010g03460	Forward	ATCATCCTTATCTGTATTCTTCT	56
	Reverse	CTTCGCCGTCACCATTCT	
VIT_202s0154g00070 (*VviYABBY*)	Forward	TCTGCCAATGTCGGTGTCTC	50
	Reverse	CGAAGCATAAAGGATAAGTC	
VIT_202s0033g01400	Forward	GGAGAGTTGAGTCTGGTGC	55
	Reverse	TTGTGAGTTACTGGGGCG	
VIT_207s0104g01810	Forward	AGGCACAATGACCTCAAAGAAG	63
	Reverse	TCGGTTTCTGAGCAGTGTGGCT	
VIT_215s0046g03160	Forward	AAGCCTGGTCGTGGTGAT	61
	Reverse	ATCAGACAAACTAAAGAGTCCC	
VIT_215s0046g02560	Forward	GAAATCTTCCTTTGTTCCTTGT	60
	Reverse	TATTCTTTGTTGTGTTTGTGGC	
